# Supplementary material for: Understanding the mental health impact and needs of public healthcare professionals during COVID-19 in Pakistan : a qualitative study
Source: BMJ Open. 2022 Nov 7;12(11):e061482. doi: 10.1136/bmjopen-2022-061482 (PMC9644080; doi:10.1136/bmjopen-2022-061482)
Supplement: Supplementary data [file bmjopen-2022-061482supp002.pdf]

BMJ Publishing Group Limited (BMJ) disclaims all liability and responsibility arising from any reliance placed on this supplemental material which has been supplied by the author(s)

COVID-19: psychosocial and mental health needs of health workers during the outbreak management in Pakistan

IN-DEPTH INTERVIEW GUIDE FOR HEALTH MANAGERS AND SERVICE PROVIDERS

S.no	Questions	Responses	Skip
**SECTION 1: PARTICIPANT CONTACT SHEET**			
Please complete before the interview.			
101	Name of province	Sindh 1<br>Punjab 2	
102	Name of district		
103	Name of TALUKA / Tehsil		
104	Name of Union Council		
105	Type of health facility	Basic Health Unit (BHU) 1<br>Rural Health Centre (RHC) 2<br>Taluka Head Quarter (THQ) 3<br>District Head Quarter (DHQ) 4<br>Tertiary care hospital 5<br>Specialised Isolation Centre 6	
106	Name of health facility / Hospital		
107	Date of interview	______ / _______ / _____________<br>dd mm yyyy	
**SECTION 2: PARTICIPANTS PROFILE**			
201	Sex	Male 1<br>Female 2	
202	Marital status	Never married 1<br>Currently married 2<br>Divorced 3<br>Separated 4<br>Widowed 5	
203	Age (in years)	Years [ ][ ]	
204	Number of living children	Children [ ][ ]	
205	Do you live in a nuclear family or joint family?	Nuclear 1<br>Joint 2	
206	Highest level of education completed?	Matriculation 1<br>Intermediate 2<br>Bachelors 3<br>Masters / M.Phil. 4<br>PhD 5	
207	Professional qualification	Lady Health Visitor 1<br>Lady Health Worker 2	

Version 1.0 Last updated 29th June 2020

Hameed W, *et al. BMJ Open* 2022; 12:e061482. doi: 10.1136/bmjopen-2022-061482

		Midwife 3<br>Nurse 4<br>M.B.B.S 5	
208	What is the current position you hold in the organization?	Lady health supervisors (LHSs) 1<br>Managers from secondary level health facility (DHQ/THQ) 2<br>Medical superintendent of tertiary care teaching hospital 3<br>Specialist from Field isolation center, Karachi 4<br>Others specify______________________ 5	
209	How long you have been working in this health facility / catchment area?	Years [ ][ ]	
210	What is the total years of your professional experience?	Years [ ][ ]	

Version 1.0 Last updated 29th June 2020

Hameed W, *et al. BMJ Open* 2022; 12:e061482. doi: 10.1136/bmjopen-2022-061482

No.	Questions	Responses	Probe
**1**	**THEME : Understanding psychological impact of covid-19 on healthcare workers**		
	**First, I would like to ask you some questions to understand the psychological impact of covid-19 on healthcare workers**<br>**The psychological adverse outcomes may include stress, anxiety, depression, burnout, post-traumatic stress disorder, fear-triggered panic attacks, psychosomatic symptoms, psychological violence, fatigue, stigma etc.**		
1.1	How healthcare workers feel about working under tremendous pressure with COVID -19 situation?		Probes:<br>• Can you use example to explain the situation?
1.2	In your opinion, how has COVID-19 affected mental health of healthcare workers?		Probes:<br>• How healthcare workers are dealing with anxiety and fear related to managing COVID-19 inpatients?<br>• Is it affecting healthcare workers both negatively and positively?
1.3	Can you specify the reasons for adverse psychological outcomes among healthcare workers?		Probes:<br>• Reasons related to procedural changes (increased workload, long working hours)
1.4	How negative emotions among healthcare workers will affect the overall functioning of organisations/ institutions?		Probes:<br>• Do you think negative emotions among health worker may lead to poor health worker attention, understanding and decision-making ability
1.5	Do you think health workers have capacity to cope with psychological risk?		Probes:<br>• If yes, what are the coping mechanisms of healthcare workers?<br>• If no, why do you think healthcare workers lack capacity to cope with psychological risk?
1.6	Do you think psychological needs of population has changed in recent times?		Probes:<br>• How health system is responding to those psychological needs?<br>• Are health workers prepared to address changing psychological needs of patients with covid-19?
**2**	**THEME: Identifying psychological needs of health workforce involved in covid-19 crisis**		
	**Now I would like to ask some questions to understand the psychological needs of health workforce involved in covid-19 crisis. The psychological needs of healthcare workers may include enabling working environment, safe and secured hospital environment, adequate staffing, support from mentors and floor managers, support from mental health counsellors, etc.**		
2.1	What are the psychological needs of health workforce involved in covid-19 crisis		Probes:<br>• How did you get to know about their psychological needs?

Hameed W, *et al. BMJ Open* 2022; 12:e061482. doi: 10.1136/bmjopen-2022-061482

2.2	Do you think healthcare workers are able to talk about their psychological needs and concerns?		Probes:<br>• If yes, please share examples of some instances where healthcare workers have shared their psychological needs<br>• If no, why healthcare workers are not able to talk about their concerns?
2.3	As a _____, how do you see your role in identifying the psychological needs of the health workforce		
**3**	**THEME: Support mechanisms available to address mental health needs of healthcare workers**		
	I would like to know the support mechanisms/ processes available to address mental health needs of healthcare workers. These support mechanisms may include providing mental health support services, delivering counselling sessions, supporting workers through mental health helpline, etc.		
3.1	What are the mechanisms available to support the mental health needs of healthcare workers?		Probes:<br>• What interventions are currently used to support the mental health needs of healthcare workers?<br>•
3.2	Do you think healthcare workers are able to receive adequate mental health support with these processes?		Probes:<br>• Are they effective? Are these interventions appreciated by current health workforce?
3.3	Do you think your institution/health facility is prepared to manage mental health needs of the healthcare workers?		Probes:<br>• If yes, please share a recent experience whereby mental health needs of a health worker were adequately addressed.<br>• Do you think health system is currently prepared to address the mental health needs of health care workers<br>○ If yes, why? If no, why?
3.4	Have you received any formal training/ session on supporting mental health needs of the healthcare workers?		Probes:<br>• How many times the training was conducted and for whom?<br>• Can you tell me the objective of each training?<br>• What aspects were covered in the training?<br>• Who conducted those trainings?<br>• What was the duration of each training?
3.5	As a _____, how do you see your role in addressing the psychological needs of the health workforce		Probes:<br>• How do you provide the support to your staff?<br>• Do you feel confident while addressing the mental health needs of your staff?

Hameed W, *et al. BMJ Open* 2022; 12:e061482. doi: 10.1136/bmjopen-2022-061482

			• How do you feel about your capacity in terms of addressing psychological needs of your staff<br>• How effective it is?
4	**Theme: Understanding challenges in addressing mental health needs of healthcare workers**		
	Now I would like to understand the challenges you face while addressing mental health needs of health care workers like lack of adequate trainings, tools and resources		
4.1	What are some of the barriers you face in addressing mental health needs of healthcare workers?		Probes:<br>• Do you think lack of trainings/resources as one of the barriers to addressing mental health needs of healthcare workers?<br>• How health system can address these barriers?
4.2	In your opinion, how these barriers could be handled efficiently, to ensure adequate mental health support for healthcare workers?		
5	**Theme: Suggestions and recommendations to address psychological needs of health care workers**		
	Here I would like to understand how institutions could provide mental health support to healthcare workers. Specifically, we would like to understand the interventions that the health system can offer to alleviate psychological burden of healthcare workers.		
5.1	What kind of relief healthcare workers expect to manage with these pressures?		
5.2	Do you have suggestions on how institutions could provide mental health support services for coping with stress related to this pandemic		Probes:<br>• How health system is prepared to address psychological needs of healthcare workers?<br>• How health system can ensure implementation of process and tolls to support mental health needs of healthcare workers?<br>• How health system will sustain the mental health support activities for its healthcare workers?
5.3	Do you have suggestions on how provincial/federal government could provide mental health support services for coping with stress related to this pandemic		

Hameed W, *et al. BMJ Open* 2022; 12:e061482. doi: 10.1136/bmjopen-2022-061482
