# Supplementary material for: Understanding the mental health impact and needs of public healthcare professionals during COVID-19 in Pakistan : a qualitative study
Source: BMJ Open. 2022 Nov 7;12(11):e061482. doi: 10.1136/bmjopen-2022-061482 (PMC9644080; doi:10.1136/bmjopen-2022-061482)
Supplement: Supplementary data [file bmjopen-2022-061482supp003.pdf]

Table S1: Sample process of conventional content analysis

Text from transcript	Step 1: Readings transcripts and formulating open codes	Step 2: Developing Codebook	Step 3: Combined codes into categories	Step 4: Combine categories into themes
They (HCPs) were afraid for their families that our parents are old and if we carry coronavirus from here then we might not get them infected. Even if we don't show any symptoms, we would get them infected or we would get our children infected. So this fear was also prevailing	Infection to old parents<br><br>Infection to children	Fear of infection	Fear of of infection, isolation and stigma	Psychological impact of Covid-19 on health service providers
Immediately after that, there was fear of isolation, like if you put yourself into isolation who will look after your family and your kids …. Health workers were hospital-bound and were afraid of living alone and being cut off from their families. They also felt the stigma from the people around them	Afraid of living alone<br><br>No one to look after family<br><br>Stigma from the people	Fear of Isolation<br><br>Fear of Stigma		
Firstly, the PPEs we were using, we had to use them twice or thrice. this was our main issue. We had to wear the mask for two to three days even one week continuously<br><br>One can call it anxiety, you can see it, and they were under a depressive state. All healthcare workers were. From doctors to nursing staff, paramedics, our sweepers, all our workers were going through depression	Reuse PPEs<br><br>Workers going through depression	Lack of availability of PPEs<br><br>Depression due to COVID-19	Stress due to poor availability of personal protective equipment<br><br>Anxiety due to uncertainty of Covid-19	
