# Supplementary material for: Understanding the mental health impact and needs of public healthcare professionals during COVID-19 in Pakistan : a qualitative study
Source: BMJ Open. 2022 Nov 7;12(11):e061482. doi: 10.1136/bmjopen-2022-061482 (PMC9644080; doi:10.1136/bmjopen-2022-061482)
Supplement: Author's manuscript [file bmjopen-2022-061482.draft_revisions.pdf]

BMJ Open

BMJ Open is committed to open peer review. As part of this commitment we make the peer review history of every article we publish publicly available.

When an article is published we post the peer reviewers' comments and the authors' responses online. We also post the versions of the paper that were used during peer review. These are the versions that the peer review comments apply to.

The versions of the paper that follow are the versions that were submitted during the peer review process. They are not the versions of record or the final published versions. They should not be cited or distributed as the published version of this manuscript.

BMJ Open is an open access journal and the full, final, typeset and author-corrected version of record of the manuscript is available on our site with no access controls, subscription charges or pay-per-view fees (http://bmjopen.bmj.com).

If you have any questions on BMJ Open's open peer review process please email info.bmjopen@bmj.com

BMJ Open

Health Systems Response and COVID-19: Psychosocial and Mental Health Needs of Healthcare Professionals in Pakistan

Journal:	*BMJ Open*
Manuscript ID	bmjopen-2022-061482
Article Type:	Original research
Date Submitted by the Author:	27-Jan-2022
Complete List of Authors:	Hameed, Waqas; The Aga Khan University, Community Health Sciences<br>Feroz, Anam Shahil; Aga Khan University, Department of Community Health Sciences<br>Avan, B; London School of Hygiene and Tropical Medicine,<br>Khan, Bushra; University of Karachi, Department of Psychology<br>Fatmi, Zafar; Aga Khan University, Department of Community Health Sciences<br>Afzal, Noreen; Aga Khan University, Department of Community Health Sciences<br>Jafri, Hussain; Fatima Jinnah Medical University<br>Wassan, Mansoor Ali; Health Department Government of Sindh<br>Siddiqi, Sameen; Aga Khan University Medical College Pakistan, Community Health Sciences Department
Keywords:	COVID-19, MENTAL HEALTH, PSYCHIATRY

SCHOLARONE™
Manuscripts

I, the Submitting Author has the right to grant and does grant on behalf of all authors of the Work (as defined in the below author licence), an exclusive licence and/or a non-exclusive licence for contributions from authors who are: i) UK Crown employees; ii) where BMJ has agreed a CC-BY licence shall apply, and/or iii) in accordance with the terms applicable for US Federal Government officers or employees acting as part of their official duties; on a worldwide, perpetual, irrevocable, royalty-free basis to BMJ Publishing Group Ltd ("BMJ") its licensees and where the relevant Journal is co-owned by BMJ to the co-owners of the Journal, to publish the Work in this journal and any other BMJ products and to exploit all rights, as set out in our licence.

The Submitting Author accepts and understands that any supply made under these terms is made by BMJ to the Submitting Author unless you are acting as an employee on behalf of your employer or a postgraduate student of an affiliated institution which is paying any applicable article publishing charge ("APC") for Open Access articles. Where the Submitting Author wishes to make the Work available on an Open Access basis (and intends to pay the relevant APC), the terms of reuse of such Open Access shall be governed by a Creative Commons licence – details of these licences and which Creative Commons licence will apply to this Work are set out in our licence referred to above.

Other than as permitted in any relevant BMJ Author's Self Archiving Policies, I confirm this Work has not been accepted for publication elsewhere, is not being considered for publication elsewhere and does not duplicate material already published. I confirm all authors consent to publication of this Work and authorise the granting of this licence.

For peer review only

Title:
Health Systems Response and COVID-19: Psychosocial and Mental Health Needs of Healthcare Professionals in Pakistan

Short running title
COVID-19 and mental health needs of healthcare professionals

Authors:
Waqas Hameed[1], Anam Shahil Feroz[1], Bilal Iqbal Avan[2], Bushra Khan[3], Zafar Fatmi[1], Noreen Afzal[1], Hussain Jafri[4], Mansoor Ali Wassan[5], Sameen Siddiqi[1]

[1] Department of Community Health Sciences, Aga Khan University, Karachi, Pakistan
[2] Department of Clinical Research, London School of Hygiene and Tropical Medicine, London, United Kingdom
[3] Department of Psychology, University of Karachi, Sindh, Pakistan
[4] Fatima Jinnah Medical University, Lahore, Pakistan
[5] Department of Health, Government of Sindh, Pakistan

Correspondence:
Bilal Iqbal Avan
Associate Professor
Department of Clinical Research
London School of Hygiene and Tropical Medicine
Keppel St, London WC1E 7HT, United Kingdom
bilal.avan@lshtm.ac.uk

For peer review only

Abstract:

Introduction:

Dearth of qualitative studies constraint in-depth understanding of health service providers' perspectives and experiences regarding the impact of COVID-19 on their mental health. This study explored the psychological impact of COVID-19 as well as mental health needs of public-sector healthcare workers in secondary- and tertiary-level healthcare settings of Pakistan.

Methods:

We conducted an exploratory qualitative study using a criterion purposive sampling approach, at twenty-five secondary- and eight tertiary-level public sector hospitals of the Sindh and Punjab provinces of Pakistan. In-depth interviews were conducted with sixteen health service providers, and forty administrative personnel. Study data were analysed on NVivo 11 using the conventional content analysis technique.

Results:

The study identified three overarching themes: (I) Psychological impact of covid-19 on health service providers that included the fear of acquiring infection and transmitting it to their family members, fear of social isolation and stigma, anxiety related to the uncertainty of Covid-19, nervousness due to media exaggeration, and stress associated with excessive workload; (II) Psychological needs of health service providers involved in COVID-19 crisis and available support from healthcare system. They expressed the need for counselling services and safe working conditions, the need for paid rest periods, and the need for appreciation and motivation to work in the pandemic; and (III) Suggestions to address mental health needs of healthcare workers were: provision of specialized mental health care/services, conducting formal training of health managers on managing mental health needs of health facility staff, and assess mental health needs of the health workforce and act accordingly.

Conclusion:

The study emphasises the need to strengthening health system preparedness for recognizing and addressing personal and professional needs of health professionals through establishing psychological health services; motivating and appreciating health service providers; and providing extra rest periods and incentives to compensate for the additional risks associated with Covid-19.

Keywords: COVID-19, corona virus, mental health, healthcare workers, psychological

For peer review only

Strengthens and limitation of the study:

- This study not only provides an in-depth experiences of health care professional regarding the psychological impact of COVID-19, but also their mental health needs, and suggestions to address their psychological in pandemic crisis
- Unlike urban-focused studies, the participants in this study were invited across twenty-two urban and rural districts in two provinces of Pakistan
- One key limitation was that the interviews were conducted on phone owing to the restricted mobility during COVID-19. They may introduce some risk of bias as the interviewers did not have the opportunity to build rapport as in the case of face-to-face interviews

Introduction

Worldwide, health service providers are under insurmountable psychological pressures as they are expected to deal with a number of issues that arise due to the Covid-19 situation[1]. First, due to the increased clinical workload associated with Covid-19 cases, health service providers face increasingly long work hours, often with limited resources and weak infrastructure[2]. Second, they face physical discomfort associated with donning and doffing of personal protective equipment (PPE), or the unavailability of it, which is essential to keep them safe from exposure to the virus [3]. Third, health service providers feel unprepared to deal new and unfamiliar task since the evidence-based clinical treatments for Covid-19 continue to evolve [3] . Finally, there is a very valid fear of autoinoculation and the risk of transmitting the virus to family members [4,5] Unsurprisingly, all these factors take a toll on the mental health of healthcare professionals (HCPs) and result in various psychiatric issues including fear [6], anxiety [1], depression [1], burnout [7], trauma [8], and insomnia [9].

Evidence show that psychological health implications for health workers who are involved in the provision of healthcare during epidemics and pandemics are long-lasting [10,11]. For instance, healthcare providers suffered from dysphoria and stress in 2015 during the outbreak of the Middle East respiratory syndrome (MERS) [10]. Moreover, experiences of Evidence suggests that high levels of stress, anxiety, depression, and even post-traumatic stress disorder (PTSD) have been observed in many cases even after some time had transpired after such outbreaks [11]. Therefore, it is extremely important to understand the psychological impact of Covid-19 on health service providers as well as the psychological needs of health service providers who are involved in healthcare provision at the hospital level.

To date, a number of quantitative research studies have been conducted to report the magnitude of adverse psychological outcomes faced by health service providers who are directly involved in the diagnosis, treatment, and care of Covid-19 patients [1,3,6,8]. These quantitative studies are limited in providing an in-depth understanding of health service providers' perspectives and experiences regarding the impact of Covid-19 on the mental health of health workers. There is the paucity of qualitative evidence to understand the health system response regarding the psychological impact of Covid-19 on health service providers, which is essential to develop context-specific mental health interventions to support the psychological health of the current health workforce. A qualitative study was conducted in England to explore experiences and concerns of front-line National Health Service (NHS) workers while caring for patients with COVID-19. The study concluded that Covid-19 patients brought a significant emotional toll, and strained relationships between immediate front-line staff, and their families. At the beginning of the pandemic, staff were driven by adrenalin and optimism; but over time this dissipated to be replaced by exhaustion, numbness, and dreadful expectation of a ‘second wave’ [12]. Another

For peer review only

qualitative study explored the psychological impact of Covid-19 on emergency health service providers working in public sector hospitals of Pakistan [13]. This study presented a thorough understanding of how the frontline emergency health care workers are dealing with the Covid-19 pandemic, their stress-coping strategies or protective factors, and challenges while dealing with Covid-19 patients.

These studies are limited in providing evidence on health systems' preparedness to respond to the mental health needs of health service providers from the perspective of health managers and health service providers. Prior studies have shown that system-level mental health interventions during the SARS epidemic were effective in reducing health service providers' symptoms of anxiety and depression [1-4,11]. Such evidence necessitates that the health system preparedness and response are needed to cater to these needs to prevent the deterioration of health service providers' mental health during Covid-19. This study is the first to explore the psychological impact of Covid-19 as well as the mental health needs of health service providers in public healthcare settings of Pakistan from the perspective of health managers and health service providers including doctors and nurses.

Our study aims to answer the following research questions:

- What is the perceived psychological impact of the Covid-19 pandemic on the performance of health service providers in secondary and tertiary care settings in Pakistan?
- What are the health service providers' psychological and mental health needs working amid the Covid-19 pandemic, and how well the health system is responding to them?
- What are the recommendations by the health service providers to address their psychological and mental health needs?

Materials and Methods

Consolidated criteria for reporting qualitative research (COREQ) checklist has been used for designing this qualitative study[14].

Study design and setting

This formative research employed an exploratory qualitative research design using semi-structured interviews and a criterion purposive sampling approach. Healthcare in Pakistan is delivered through a three-tiered health system. The primary health care provides evidence based essential health services through a community-based component through frontline health workers and health facility component that include Basic health units, rural health centres, maternal and child centres, and civil dispensaries. Secondary-level healthcare facilities concerns with provision of technical, therapeutic and diagnostic services. Specialist consultation and hospital admissions fall into this category. It includes Tehsil Head Quarter (THQs) and District Headquarter Hospitals (DHQs). Thirdly, tertiary healthcare hospitals are for more specialized inpatient care supported by availability of required resources. With few exceptions, these are also affiliated with the medical teaching institutions for graduates and post-graduates [15]. Our study was conducted at secondary- and territory-level public sector hospitals of Sindh and Punjab provinces of Pakistan. A total of eight teaching hospitals, eight DHQs, and twenty-five THQs were randomly selected from Sindh and Punjab Province.

Data Collection Methods and study participants

The data collection methods for this formative research included in-depth interviews (IDIs) with health service providers (doctors, nurses), head of department (HoD), and medical

For peer review only

superintendent (MS). In terms of participants' involvement in the care of Covid-19 patients, health managers including MS and HoD was indirectly involved with the management of Covid-19 patients; however, health service providers including doctors and nurses were directly involved in the care of Covid-19 patients. For secondary-level health facilities, we contacted the MS who was invited to participate in the study. Moreover, s/he was further asked to nominate service providers from their respective health facilities who may or may be not involved in the provision of care to COVID19 patients. Similarly, for teaching hospitals, we contacted the administrative head who identified a focal person on his/her behalf for further coordination. Similar, request was made to the focal person for identification of department heads and service provider.

Eligibility criteria

Health managers and health service providers employed in public sector hospitals of Sindh and Punjab providence and dealing directly or indirectly with Covid-19 patients were eligible to participate in the study. Health service providers and hospital management either belonging to private sector hospitals or from provinces other than Sindh and Punjab were excluded. Non-clinical staff such as janitors and technicians were also excluded from the study.

Data Collection Method

<u>In-depth Interviews</u>

Adapting to the Covid-19 situation, a total of 56 (Sindh: 34; Punjab:22) semi-structured telephonic IDIs were conducted in the local language (Urdu), by data collectors trained in qualitative research. The interviews are conducted at Aga Khan University Hospital under close supervision of research investigators. Keynotes were also taken by data collectors during the interviews. The IDIs were conducted until data saturation was achieved [16]. Data collection and data analysis were conducted iteratively to determine the point of data saturation [17,18]. Consistent with Creswell and Poth's [17] guidelines on interviewing, all IDIs lasted approximately between 45-60 mins. The interviews were audio-recorded on tablets with consent from study participants, which were later transcribed in the English language by a transcriptionist. The data collection took place between August and October 2020.

Interview Guide

A semi-structured interview guide was designed for IDIs through extensive literature review and consultative approach seeking expert opinions. This guide was developed to interview all research participants. The guide includes five major themes: a) Psychological Impact of Covid-19 on health service providers; b) Psychological needs of health service providers involved in Covid-19 crisis; c) Support mechanisms available to address mental health needs of health service providers; d) Challenges in addressing mental health needs of health service providers; e) Recommendations to address psychological needs of health service providers. A free flow of information was encouraged, using probes from the participants verbatim to understand their perception of mental health needs during Covid-19.

The interview guide was used flexibly to allow participants to construct their accounts on their own terms. The guide was pilot tested with a non-study sample who share the same characteristics as the study sample [17]. The pilot testing provided evidence-based direction to improve the interview guide questions 6418 [18].

Data analysis

All study data including notes, recordings, and transcriptions were uploaded into the NVivo version 11.0 software for data analysis [19]. The analysis looked at convergent and divergent lines of inquiry from the two main study groups, that is, health managers and health service providers (doctors and nurses). All qualitative data including were analysed using conventional content analysis [20]. Inductive coding was applied to transcripts to document in vivo codes and new emerging codes and categories that fall outside themes identified in the interview guide. In vivo codes are essential to identify specific vernacular used by participants' to describe the psychological impact of Covid-19[21]. A codebook was developed based on the initial codes identified during analysis in the interview guide, which was used by two researchers (AS & NA) to process, and code the data. The discrepancies in coding were resolved through a discussion with a senior researcher. Throughout the analysis, the researcher made reflective notes (memos) to document important learnings from the data [22]. AS and NA independently performed coding to ensure that interpretations are coherent, plausible, and grounded in the study data [20].

Ethical considerations
Ethical approval for this study was obtained from the Aga Khan University Ethical Review Committee (AKU-ERC) – [2020-5186-11602], and National Bioethics Committee of Pakistan – [4-87/COVID-45/NBC/20/393]. The interviews were conducted telephonically. Verbal informed consent was taken from the study participants. The audio recordings were stored in a password-protected folder accessible only to the research team. Permission letters from the Health Departments were issued by the respective provincial governments. These letters along with the consent forms were sent through WhatsApp to the District Health Officers (DHOs) of all the selected districts. Participants were also sent an SMS explaining to them the purpose of the study.

Patient and public involvement
We sought inputs from target audience through pre-testing of interview guides to ensure comprehension of the questions. Furthermore, in consultation with provincial health departments we selected districts where COVID-19 cases were high. The focal person in each selected district was oriented about the study who connected our team with in-charge of health facility to avoid any miscommunication. Lastly, they also advised about the dissemination of research findings – that is, they specifically emphasis to share it through research briefs to reach wider audience in the country.

Results

A total of 56 semi-structured IDIs were conducted to explore the psychosocial and mental health needs of health service providers amidst Covid-19 in Pakistan. Of 56 IDIs, 16 IDIs were conducted with health service providers, and 40 IDIs were conducted with health managers. All the participants (n = 56) who were approached by the study team agreed to participate. The demographic information for all the study participants is illustrated in Table 1.

Table 1: Characteristics of study participants

**Characteristics of study participants**		**N (%)**
Gender (n=56)	Female	9 (16.1)
	Male	47 (83.9)
Designation (n=56)	Hospital Managers	40 (71.4)
	Health Service Providers	16 (28.5)
Facility Type (n=56)	Secondary healthcare hospitals	37 (66.1)
	Tertiary healthcare hospitals	19 (33.9)

For peer review only

Age (n=48)	Mean	46.6
	Median	47
	SD	10.6
Experience (n=53)	Mean	15.4
	Median	15
	SD	9.5

Based on the directed content analysis, three overarching themes were identified: (I) Psychological impact of covid-19 on health service providers; (II) Psychological needs of health service providers involved in COVID-19 crisis and available support from health system; and (III) Suggestions to address mental health needs of health service providers. These themes and their categories are presented in Table 2.

Table 2: Themes and sub-themes

Themes	Sub-themes
Theme 1: Psychological impact of Covid-19 on healthcare professionals	1.1 Fear of infection, isolation and stigma<br>1.2 Stress due to poor availability of personal protective equipment<br>1.3 Anxiety due to uncertainty of Covid-19<br>1.4 Anxiety and nervousness due to media exaggeration<br>1.5 Excessive workload
Theme 2: Psychological needs of health workforce involved in Covid-19 crisis and available support from health system	2.1 Need to provide counselling services and safe working conditions<br>2.2 Need for paid rest periods, timely payment of workers' salaries, and incentives<br>2.3 Need of appreciation and motivation to work in the pandemic
Theme 3: Suggestions to address the mental health needs of healthcare professionals.	3.1 Establish mental health services to address the psychological needs of health workers<br>3.2 Conduct formal training of health managers on managing mental health needs.<br>3.3 Assess and address mental health needs of the health workforce

1. Psychological impact of Covid-19 on health service providers

1.1 Fear of infection, isolation and stigma

The interviews with hospital managers and health service providers revealed that all health service providers felt fearful of acquiring the Covid-19 infection and transmitting the infection to their family members. A hospital manager mentioned that health service providers are particularly concerned about their parents and children who are vulnerable and might get infected.

For peer review only

"They (HCPs) were afraid for their families that our parents are old and if we carry coronavirus from here then we might not get them infected. Even if we don't show any symptoms, we would get them infected or we would get our children infected. So this fear was also prevailing." (Hospital Manager, Punjab)

Health service providers were fearful that if they did acquire the infection, they would have to isolate themselves from their families. Many workers could not afford to isolate themselves in the event they tested positive for Covid-19.

"They used to say that if the report comes back positive, then how they will stay at home because their homes are small" (Hospital Manager, Sindh)

"Immediately after that, there was fear of isolation, like if you put yourself into isolation who will look after your family and your kids". (Hospital Manager, Punjab)

Both groups of respondents highlighted that few health service providers resigned from their jobs since their families were getting infected because of them. In particular, female health service providers had higher pressure on them from their families to leave their jobs. While some providers refused to work in Covid-19 wards, others threatened health managers to commit suicide, if they were posted there. Health managers verbalized that a lot of the health service providers posted in Covid-19 wards revealed the fear of being isolated and stigmatized.

"Health workers were hospital-bound and were afraid of living alone and being cut off from their families. They also felt the stigma from the people around them". (Hospital Manager, Sindh)

1.2 Stress due to poor availability of personal protective equipment

A major concern among the health service provider was the lack of protective gear available to them. So, treating Covid-19 positive or suspected cases without PPE was escalating their fear of getting infected. They felt that they had been sent to fight a war without weapons.

"Firstly, the PPEs we were using, we had to use them twice or thrice... this was our main issue. We had to wear the mask for two to three days even one week continuously" (Health Service Provider, Punjab)

On the other hand, health managers revealed that some health service providers labelled them as being insensitive and unaware of the problems faced by workers due to lack of PPE availability. However, managers themselves were helpless due to its lack of availability in the market and ended up buying masks and other protective equipment for the staff themselves. But this protective gear was not up to the mark.

"That time if anyone didn't get PPE they (health service providers) would get hyper but we provided PPEs at our facility. We would provide requisite equipment soon so we didn't have to face so much trouble." (Hospital Manager, Punjab)

1.3 Anxiety due to uncertainty of Covid-19

Health service providers reported feeling extremely anxious working with Covid-19 patients. Owing to the novelty and unfamiliarity of the virus along with workers being inadequately equipped with the required knowledge about the disease, they were uncertain about the consequences. Health service providers were also having trouble identifying cases of Covid-19 since there were both, symptomatic and asymptomatic cases. They were not clear about the mode of transmission of the virus.

For peer review only

“One can call it anxiety, you can see it, and they were under a depressive state. All healthcare workers were. From doctors to nursing staff, paramedics, our sweepers, all our workers were going through depression.” (Health Service Provider, Sindh)

Some hospital managers had contrasting views about the difficulties faced by their staff while working during the pandemic. They claimed that health service providers were not stressed while working during the COVID-19 pandemic and that none of them came to the managers to express their concerns.

“I don’t think their psychological health was affected, I don’t think so, and there was stress only in the beginning when they were not aware of the atmosphere” (Hospital manager, Sindh)

“No there was no pressure on them, we were doing many meetings, we were explaining it to them, that there’s no issue, this time has come we will solve it Insha’Allah, we will be successful, there wasn’t an issue like this in our staff, they were definitely cooperating with us, that’s why there was no stress or any problem like that” (Hospital manager, Sindh)

1.4 Anxiety and nervousness due to media exaggeration

Both groups of respondents reported that media has a strong role in mounting anxiety and distress among healthcare workers. The media was seen to exaggerate the magnitude of the situation and there was a bombardment of information. News regarding deaths of workers due to Covid-19 was also creating panic among the majority of the healthcare workers.

“But this novel coronavirus made hype through digital media as no one knew what it was as initially people were stressed” (Hospital Manager, Sindh)

False information was seen to circulate about the negative consequences for people testing positive for Covid-19, which was creating a lot of distress among healthcare workers currently working in Covid-19 wards. In addition, news related to the long-term complications of Covid-19 increased nervousness among healthcare workers.

“If you take my personal opinion, the hype created regarding this is too much, that has spread too much tension. That has bothered people a lot about what will happen, God forbid if somebody has corona then he will be taken away or this will happen or that will happen, etc. or because of me, my children will suffer”. (Health Service Provider, Punjab)

1.5 Excessive workload

Both groups of respondents mentioned that health service providers felt strained and exhausted because of the clinical workload. Due to the shortage of health workforce, the existing pool of health service providers had to bear the burden of extra clinical work. Their duty hours increased along with the number of shifts they had to do per week, which resulted in increased psychological distress among health service providers.

“So far, no one told me that their mental health has been affected. …yes, some people have said that they are depressed…they are feeling tired and have motion sickness due to extra working hours”. (Health Manager, Punjab)

The Covid-19 outbreak among health service providers led to further staff shortages and increased mental pressure among health service providers. Both groups of respondents

For peer review only

mentioned that even after returning from sick leave, health service providers were not performing at their best. Some of them seen to become weak and some of them would faint. In addition, some workers' tests were negative for the Covid-19 infection, but they still had cough symptoms which would make their co-workers anxious. Hence, they also had to be given leaves.

"As the work load increased, so did the pressure on the HCWs and their efficiency began to decrease". (Health Manager, Sindh)

Both groups of respondents mentioned that their commute time to the hospital also significantly increased due to the unavailability of public transport during the lockdown which further increased the psychological burden among health service providers.

"Our doctors faced a lot of transport issues but we tried our best to support them. Few of our staff were punctual initially, but some of them were not coming at all due to fear of COVID-19. Their wages are less so we reduced the duration of their duties and counselled them a lot after which they started coming to work. They come from far off areas so they should be given space here at the hospital so that they can perform their duties easily". (Health Manager, Sindh)

2. Psychological needs of health workforce involved in Covid-19D-19 crisis and available support from health system

2.1 Need to provide counselling services and safe working conditions

Health service providers and health managers highlighted the need for counselling services for health service providers working under pressure to provide care to Covid-19 patients. Health service providers mentioned the desire to receive one-to-one counselling sessions from mental health professionals to reduce the stress and anxiety they were experiencing because of working during Covid-19.

All hospitals should at least provide one-to-one …counselling sessions…to reduce staff anxiety and stress. (Health Service Provider, Sindh, male)

Since no proper counselling services from mental health professionals were available, informal counselling to health service providers was provided by hospital managers. Although the managers were trying to support their staff, they lacked formal training in providing counselling. They motivated them via reinforcing health providers' professional and moral obligations but it did not prove fruitful for most of the workers.

"Whatever knowledge I had regarding the disease and its spread, and I also told them that we must serve humanity, and death is inevitable and I did whatever I could, regarding the moral values, whatever I could make them understand, some of them understood and most of them didn't". (Hospital Manager, Sindh)

"I told them with a passion that the death Allah has written, if we die on this job, they will be martyred" (Hospital Manager, Punjab)

Health managers from few facilities reported that the government had arranged webinars for health service providers to help them manage psychological stress. However, the effectiveness of these training was quite low because many health workers had duties due to which they were unable to attend or sit through them.

For peer review only

In addition, health service providers highlighted the need of working in a safe and secure working environment. They further mentioned that the lack of safety was largely attributed to the inadequate supply of protective equipment. To make up for the lack of PPEs, health service providers resorted to using the same protective gear they had repeatedly for multiple days.

"In many places, we have seen that doctors and paramedical staff were affected and all this is due to the lack of what was needed, for example, the lack of hand sanitizers and masks". (Hospital Manager, Sindh

2.2 Need for paid rest periods, timely payment of workers' salaries, and incentives

Health service providers also highlighted the need for extra rest periods so that they could get enough time to relax from the exhaustive routine and then come back to work. A common strategy employed by the health managers was to give short leaves to the health service providers most affected by the COVID-19 stress. These leaves helped the staff in regaining their mental and physical strength to work.

"They (health service providers) used to say we will do work for 15 days and then require 15 days for holidays, we will relax during that time, I said ok I will give that to you". (Hospital Manager, Sindh)

In addition, the majority of health service providers and health managers reported that lack of additional allowance for staff directly dealing with COVID-19 patients was a demotivating factors especially for paramedical staff.

"The poor paramedical staff who are the first ones to interact with the patients get paid very less. The government did not do anything for them because the staff used to complain that we are getting the same salaries while having maximum exposure to COVID-19." (Health Service Provider, Punjab)

Health service providers also wanted to be given monetary incentives because working during the pandemic meant that their lives were at stake. This would also help to increase their motivation to work during such difficult conditions.

"They should be given incentive because they are keeping their life on risk" (Health Service Provider, Sindh)

2.2 2.3 Need for appreciation and motivation to work in the pandemic

Hospital managers and health service providers reported that workers needed appreciation for all the hard work they were doing. And providing appreciation would, in turn, lead to the workers becoming motivated to continue their efforts. Health service providers were putting their lives at stake while working in such difficult conditions without adequate sources, hence their efforts should be commended.

"Then they (health managers) should arrange programs in which they appreciate and motivate their employees and provide them proper guidance then it will have major positive effects on mental health". (Health Service Provider, Punjab)

For peer review only

It was also suggested that the staff should be given certificates to appreciate the work they were doing and to hold regular meetings with them to see work progress and understand their needs. Health service providers mentioned that motivating sessions are also needed to boost morale of the workers who are working in such difficult conditions.

I told my hospital director that we need a psychologist. I also highlighted that our human resource team should be given appreciation certificates other than monetary incentives which might boost their motivation to work. Meetings should also be conducted with them to ask them about their work and what else they need since this is also lacking currently. (Hospital Manager, Sindh)

3) Suggestions to address mental health needs of healthcare professionals

3.1 Establish mental health services to address the psychological needs of health workers

Both groups of respondents stressed that there needed to be an increase in the budget allocated to the health sector to establish mental health services for health service providers working during Covid-19. More funds and resources would lead to betterment for healthcare workers. Hiring mental health professionals including psychologists and psychiatrists to support the health service providers' psychologically were strongly recommended and emphasized repeatedly by health managers.

With regards to the roles of provincial governments in improving the mental health of healthcare professionals, there was a need for the implementation of formal programs addressing mental health. The Government was also advised to arrange seminars on mental health for health service providers alongside launching a mobile application for providing relevant information on the topic.

"There should be seminars and sessions and the Punjab government should launch an app regarding this so that we get information from it regarding mental and physical health and keep on updating it. This will improve the situation". (Health Service Provider, Punjab)

Hospital managers were also of the view that government officials should visit hospitals and have discussions with all levels of health service providers to understand their experiences and needs. This would enable the government to formulate effective strategies in dealing with problems faced by health service providers. Some health service providers complained that their health managers were not bothered if their staff was facing any issues.

"Nobody bothers to discuss any issue faced by workers. They only give orders to perform their duties. Our hospital managers, they don't have any concerns if workers are having any problems or not" (Health Service Provider, Sindh)

Sorting logistical issues such as providing transportation facilities for health service providers to and from quarantine centres was also stressed upon.

3.2 Conduct formal training of health managers on Managing Mental Health needs

Since hospital managers were directly overseeing the health service providers and had the most interaction with them, their formal training in providing basic counselling to the staff was deemed necessary. Health service providers wanted managers to hold daily meetings with them where they could discuss the difficulties they were facing while caring for COVID-19 positive patients.

For peer review only

“The administrator of the hospital should talk about it from every doctor. He should do meeting with them to see if they face any mental pressure or not and if he has so the problem then he should sort out his problem” (Health Service Provider, Sindh)

Some health service providers also remarked that hospital managers should be more approachable so that the staff feels comfortable in sharing their concerns with them.

“If employees are guided properly in monthly or weekly meetings, their issues are being heard whatever they are. There should be less difference of job between an employee and MS or head of the department so that he can go and discuss his problems with him, so many of problems can be solved” (Health Service Provider, Punjab)

3.3 Assess and address mental health needs of the health workforce

Another recommendation for the future was for the government and health managers to plan all the measures the health system would need to take in the event of such a crisis. They should have observed the situation in countries such as China where the outbreak first began and started thinking of strategies to get a better grip of the impending problems.

“We don’t know if this pandemic will subside or not. God Forbid if this second waves comes and traffic of patients increases, then what will we do? This way we will get depressed, so proper training should definitely be given, mental health workers should come, go to the institution and guide the HCWs.” (Health Manager, Sindh)

Health service providers also suggested that keeping in mind the difficult conditions they were eventually going to have to work in, there should have been sessions that would mentally prepare them for the latter.

“When you send a doctor in the COVID-19 ward, there should have been a week-long training with a psychologist, to explain them the situation, what will be their duties, the government has selected you, for this task, they should have been prepared and there is nothing to worry about, the mortality rate is low, and most people recover” (Health Service Provider, Sindh)

Discussion

Our study provided an in-depth investigation into the psychological impact of Covid-19 on health service providers and mental health needs of different cadres of healthcare workers in public health sector settings. Perceptions of the two groups of respondents on most issues were largely similar. There was an adverse perceived psychological impact experienced by health service providers while working during the pandemic. The available evidence also highlights that the health service providers are both a central component of the pandemic response and among those most vulnerable to infection and mental health impacts due to their professional exposure [23]. The health service providers expressed several explanations for psychological stress including the fear of acquiring infection and transmitting it to their family members, fear of social isolation and stigma, anxiety related to the uncertainty of Covid-19, nervousness due to media exaggeration, and stress associated with excessive workload. Consistent with findings of our study, the World Health Organization (2020) interim guidance document reports that the threats to health workers’ mental health during Covid-19 include the high intensity of care, increased volume of clinical services leading to overburdening, overall situational anxiety, and infection risk (compounded by lack of PPE) for health workers themselves and their families [23]. Divergent

For peer review only

views among the two groups were also found on the impact of Covid-19 on the psychological health of health service providers. It is worrisome to note that while health service providers claimed to experience significant adverse psychological effects due to Covid-19, some health managers denied that their staff faced any stress or difficulty working during the pandemic. Hence, it is important to investigate the causes of such contrasting views about the same phenomenon by different groups, especially when it affects the health system at large.

The study revealed the psychological health needs of health service providers including the need to provide counselling services and safe working conditions, the need for paid rest periods and timely payment of workers' salaries, and the need for appreciation and motivation to work in the pandemic. Previous experience from the outbreak of Ebola in western Africa highlighted the need to provide risk allowance for motivating and retaining healthcare workers [7]. Our study also highlighted the need to provide risk allowance to health service providers. In addition, our study emphasized the need to provide timely salaries to the health service providers, and extra rest periods. Both groups of respondents revealed that the health system response in the given scenario has been weak given the lack of existing support mechanisms to address the mental health needs of the health workforce during Covid-19.

Finally, the health service providers and health managers provided three suggestions to address the mental health needs of health service providers in the future including the provision of mental health services to address the psychological needs of health service providers, conducting formal training of health managers on managing mental health needs, and anticipating the mental health needs of the health workforce to guide future initiatives. Experiences from similar outbreaks suggest that early psychological intervention and the establishment of early support systems are particularly important to promote the emotional release and improve health service providers' mental health [24]. World Health Organization (2020) interim guidance document suggests that health managers should be able to perform the following five key actions to address psychological health needs of the workforce: 1) Assess and minimize additional Covid-19-related occupational psychosocial risks for stress; 2 Ensure access to and provision of mental health and psychosocial support services for health workers involved in the Covid-19 response; 3) Promote help-seeking and provide evidence-based resources on basic psychosocial skills for health workers; 4) Establish approaches to discuss challenges and dilemmas, organize schedules to include breaks, minimize other work-related stress and activate peer support; 5) Train health leads in basic psychosocial skills and the regular supportive monitoring of staff mental wellbeing, including protection from Covid-19-related stress [23]. Our study will use knowledge translation tools to share the key findings of this qualitative study with a diverse audience including policymakers, knowledge users, clinicians, researchers, health service providers, and hospital managers. Concerning future research direction, insights from the qualitative study would help in streamlining health system response in addressing the mental needs of the health workforce working in Covid-19 pandemic.

Strengths and Limitations

The study has several strengths and limitations. First, the study used multiple cadres of the health system (health service providers and health managers) which allowed researchers to look for converging and diverging lines of inquiry to identify common themes/ concepts and incongruence between data sources. Second, the primary author has applied reflexivity in all stages of designing the research to remain aware of her role and its influence on data collection, data analysis, and reporting. However, this study is also subject to limitations. First, the study was conducted with a focus on public sector hospitals in only two provinces of Pakistan (Sindh and Punjab), which would limit the transferability of the findings to other provinces [25]. Guba and Lincoln argue for the

For peer review only

concept of ‘fittingness’, which emphasizes analysing the degree to which situation studied matches other situation in which one is interested, provides a more workable way of thinking about transferability [25]. Thus, this study may provide insights for similar public hospitals across Pakistan that are interested in understanding the psychological impact and mental health needs of health service providers during Covid-19. Second, the study was unable to carry out member checking with study participants, which may have affected the validity of study findings. Lastly, the authors did not have the opportunity to build rapport and obtain no-verbal cues during interviews since the participants were interviewed telephonically due to the covid-19 situation, In addition, the interviewees might have been distracted during interviews by the activities in their environment and might have experienced fatigue due to long telephonic interviews

Conclusion

Our findings show that the health system of Pakistan was not prepared to deal with the mental health needs of health service professionals during Covid-19. This information is crucial in anticipating and preparing for tackling possible future pandemics. Health managers and health service providers shared similar concerns about working during the outbreak. Multiple recommendations were made for initiating nationwide initiatives for catering to the mental health of healthcare professionals. These include: a) a need to establish psychological health services to mitigate the detrimental effects of Covid-19 on the mental health of healthcare professionals in Pakistan alongside preparing for future pandemics; b) need to motivate and appreciate health service providers currently working under pressure to provide care to Covid-19 patients; c) need to provide extra rest periods, timely salaries to health service providers, and incentives and d) need to ensure better communication between health managers and health service providers so that the needs and concerns of the latter are heard and taken care of.

Declarations:

Ethical considerations

Ethical approval for this study was obtained from the Aga Khan University Ethical Review Committee (AKU-ERC) – [2020-5186-11602], and National Bioethics Committee of Pakistan – [4-87/COVID-45/NBC/20/393]. Verbal informed consent was taken from all study participants.

Conflicts of interest: None

Funding: This work was co-funded by the Aga Khan University, Pakistan and the World Health Organization

Contributions:

WH and BIA conceptualised the study with inputs from BK, ZF and SS. WH, BIA, BK, ZF, and AF developed interview guide. HJ and MW facilitated in conducting the study. AF and NA performed data analysis, supported by WH. WH and AF took the lead in writing the manuscript. All authors critically reviewed the manuscript and provided feedback in shaping the write-up, analysis and presentation of results. WH addressed comments from co-authors and finalized the manuscript.

Disclaimer:

The views expressed in this manuscript are those of the authors and contributors, and do not necessarily reflect those of the World Health Organization, or the organization to which the authors are affiliated.

For peer review only

Acknowledgements: We are grateful to our implementing partners: Department of Health, Government of Sindh; Primary and Secondary Healthcare Department (P&SHD) and Specialized Healthcare & Medical Education Department from Government of Punjab for extending their full their support and guidance to conduct this survey. We are also thankful to all our study participants for sparing their precious time and made themselves available for the interviews. Last but not the least, we thank our data collectors and project management team: Zafar Dehraj, Imran Sheikh, Sajid Brohi and Ghani Muhammad who managed the implementation of this study in a proficient manner during the pandemic.

Data availability statement: No additional data available

For peer review only

Reference List

1. Li Y, Scherer N, Felix L*, et al.* Prevalence of depression, anxiety and post-traumatic stress disorder in health care workers during the COVID-19 pandemic: A systematic review and meta-analysis. *PLoS ONE* 2021; 16:e0246454.https://doi.org/10.1371/journal.pone.0246454

2. Shigemura J, Ursano RJ, Morganstein JC*, et al.* Public responses to the novel 2019 coronavirus (2019-nCoV) in Japan: Mental health consequences and target populations. *Psychiatry Clin Neurosci* 2020; 2020/02/23:281-282.https://pubmed.ncbi.nlm.nih.gov/32034840

3. Huang JZ, Han MF, Luo TD*, et al.* Mental health survey of medical staff in a tertiary infectious disease hospital for COVID-19. *Zhonghua Lao Dong Wei Sheng Zhi Ye Bing Za Zhi* 2020; 38:192-195.https://doi.org/10.3760/cma.j.cn121094-20200219-00063

4. Xiang YT, Yang Y, Li W*, et al.* Timely mental health care for the 2019 novel coronavirus outbreak is urgently needed. *Lancet Psychiatry* 2020; 2020/02/04:228-229.https://pubmed.ncbi.nlm.nih.gov/32032543

5. Kang L, Li Y, Hu S*, et al.* The mental health of medical workers in Wuhan, China dealing with the 2019 novel coronavirus. *The Lancet Psychiatry* 2020; 7:e14.http://www.sciencedirect.com/science/article/pii/S221503662030047X

6. Hu D, Kong Y, Li W*, et al.* Frontline nurses' burnout, anxiety, depression, and fear statuses and their associated factors during the COVID-19 outbreak in Wuhan, China: A large-scale cross-sectional study. *EClinicalMedicine* 2020; 24:100424.https://www.sciencedirect.com/science/article/pii/S2589537020301681

7. Khasne RW, Dhakulkar BS, Mahajan HC*, et al.* Burnout among Healthcare Workers during COVID-19 Pandemic in India: Results of a Questionnaire-based Survey. *Indian J Crit Care Med* 2020; 24:664-671.https://pubmed.ncbi.nlm.nih.gov/33024372

8. Khattak SR, Saeed I, Rehman SU*, et al.* Impact of Fear of COVID-19 Pandemic on the Mental Health of Nurses in Pakistan. *Journal of Loss and Trauma* 2021; 26:421-435.https://doi.org/10.1080/15325024.2020.1814580

9. Raza A, Matloob S, Abdul Rahim NF*, et al.* Factors Impeding Health-Care Professionals to Effectively Treat Coronavirus Disease 2019 Patients in Pakistan: A Qualitative Investigation. *Front Psychol* 2020; 11:572450.https://pubmed.ncbi.nlm.nih.gov/33240162

10. Lee SM, Kang WS, Cho AR*, et al.* Psychological impact of the 2015 MERS outbreak on hospital workers and quarantined hemodialysis patients. *Compr Psychiatry* 2018; 2018/10/13:123-127.https://pubmed.ncbi.nlm.nih.gov/30343247

11. Li Z, Ge J, Yang M*, et al.* Vicarious traumatization in the general public, members, and non-members of medical teams aiding in COVID-19 control. *Brain Behav Immun* 2020; 2020/03/10:916-919.https://pubmed.ncbi.nlm.nih.gov/32169498

For peer review only

12. Bennett P, Noble S, Johnston S*, et al.* COVID-19 confessions: a qualitative exploration of healthcare workers experiences of working with COVID-19. *BMJ Open* 2020; 10:e043949.http://bmjopen.bmj.com/content/10/12/e043949.abstract

13. Munawar K, Choudhry FR. Exploring stress coping strategies of frontline emergency health workers dealing Covid-19 in Pakistan: A qualitative inquiry. *Am J Infect Control* 2021; 49:286-292.https://www.sciencedirect.com/science/article/pii/S0196655320306386

14. Tong A, Sainsbury P, Craig J. Consolidated criteria for reporting qualitative research (COREQ): a 32-item checklist for interviews and focus groups. *Int J Qual Health Care* 2007; 19:349-357.https://doi.org/10.1093/intqhc/mzm042

15. Hassan A, Mahmood K, Bukhsh HA. Healthcare System Of Pakistan. *International Journal of Advanced Research and Publication* 2017; 1:170-173.https://www.ijarp.org/published-research-papers/oct2017/Healthcare-System-Of-Pakistan.pdf

16. Fusch PI, Ness LR. Are We There Yet? Data Satur et? Data Saturation in Qualitative Research. *The Qualitative Report* 2015; 20:1408-1416.https://nsuworks.nova.edu/cgi/viewcontent.cgi?article=2281&context=tqr

17. Creswell JW, Poth CN. Qualitative Inquiry and Research Design: Choosing Among Five Approaches. Fourth Edition ed. SAGE Publishing; 2017.

18. Yin RK. Case Study Research and Applications: Design and Methods. Sixth Edition ed. SAGE Publishing; 2017.

19. NVivo qualitative data analysis software [QSR International Pty Ltd.; 2015.

20. Hsieh HF, Shannon SE. Three Approaches to Qualitative Content Analysis. *Qual Health Res* 2005; 15:1277-1288.https://doi.org/10.1177/1049732305276687

21. Breimaier HE, Heckemann B, Halfens RJG*, et al.* The Consolidated Framework for Implementation Research (CFIR): a useful theoretical framework for guiding and evaluating a guideline implementation process in a hospital-based nursing practice. *BMC Nursing* 2015; 14:43.https://doi.org/10.1186/s12912-015-0088-4

22. Birks M, Chapman Y, Francis K. Memoing in qualitative research: Probing data and processes. *Journal of Research in Nursing* 2008; 13:68-75.https://doi.org/10.1177/1744987107081254

23. World Health Organization. Health workforce policy and management in the context of the COVID-19 pandemic response: interim guidance, 3 December 2020. 3-12-2020. Geneva, World Health Organization. 10-10-2021.

Ref Type: Report

24. Sun N, Wei L, Shi S*, et al.* A qualitative study on the psychological experience of caregivers of COVID-19 patients. *Am J Infect Control* 2020; 2020/04/08:592-598.https://pubmed.ncbi.nlm.nih.gov/32334904

For peer review only

25. Donmoyer R. Generalizability and the Single-Case Study. In: Gomm R, Hammersley M, Foster P, editors. Case Study Method. SAGE publications Ltd.; 2009.

For peer review only

For peer review only

BMJ Open

Understanding the psychological impacts of COVID-19 on public-sector healthcare professionals in Pakistan and exploring their psychological support needs from the health system: a qualitative study

Journal:	*BMJ Open*
Manuscript ID	bmjopen-2022-061482.R1
Article Type:	Original research
Date Submitted by the Author:	15-Sep-2022
Complete List of Authors:	Hameed, Waqas; The Aga Khan University, Community Health Sciences<br>Feroz, Anam Shahil; Aga Khan University, Department of Community Health Sciences<br>Avan, B; London School of Hygiene and Tropical Medicine,<br>Khan, Bushra; University of Karachi, Department of Psychology<br>Fatmi, Zafar; Aga Khan University, Department of Community Health Sciences<br>Afzal, Noreen; Aga Khan University, Department of Community Health Sciences<br>Jafri, Hussain; Fatima Jinnah Medical University<br>Wassan, Mansoor Ali; Health Department Government of Sindh<br>Siddiqi, Sameen; Aga Khan University Medical College Pakistan, Community Health Sciences Department
**Primary Subject Heading**:	Public health
Secondary Subject Heading:	Mental health, Public health
Keywords:	COVID-19, MENTAL HEALTH, PSYCHIATRY

SCHOLARONE™
Manuscripts

For peer review only - http://bmjopen.bmj.com/site/about/guidelines.xhtml

I, the Submitting Author has the right to grant and does grant on behalf of all authors of the Work (as defined in the below author licence), an exclusive licence and/or a non-exclusive licence for contributions from authors who are: i) UK Crown employees; ii) where BMJ has agreed a CC-BY licence shall apply, and/or iii) in accordance with the terms applicable for US Federal Government officers or employees acting as part of their official duties; on a worldwide, perpetual, irrevocable, royalty-free basis to BMJ Publishing Group Ltd ("BMJ") its licensees and where the relevant Journal is co-owned by BMJ to the co-owners of the Journal, to publish the Work in this journal and any other BMJ products and to exploit all rights, as set out in our licence.

The Submitting Author accepts and understands that any supply made under these terms is made by BMJ to the Submitting Author unless you are acting as an employee on behalf of your employer or a postgraduate student of an affiliated institution which is paying any applicable article publishing charge ("APC") for Open Access articles. Where the Submitting Author wishes to make the Work available on an Open Access basis (and intends to pay the relevant APC), the terms of reuse of such Open Access shall be governed by a Creative Commons licence – details of these licences and which Creative Commons licence will apply to this Work are set out in our licence referred to above.

Other than as permitted in any relevant BMJ Author's Self Archiving Policies, I confirm this Work has not been accepted for publication elsewhere, is not being considered for publication elsewhere and does not duplicate material already published. I confirm all authors consent to publication of this Work and authorise the granting of this licence.

For peer review only

Understanding the psychological impacts of COVID-19 on public-sector healthcare professionals in Pakistan and exploring their psychological support needs from the health system: a qualitative study

Authors:
Waqas Hameed[1], Anam Shahil Feroz[1], Bilal Iqbal Avan[2], Bushra Khan[3], Zafar Fatmi[1], Noreen Afzal[1], Hussain Jafri[4], Mansoor Ali Wassan[5], Sameen Siddiqi[1]

[1] Department of Community Health Sciences, Aga Khan University, Karachi, Pakistan
[2] Department of Clinical Research, London School of Hygiene and Tropical Medicine, London, United Kingdom
[3] Department of Psychology, University of Karachi, Sindh, Pakistan
[4] Fatima Jinnah Medical University, Lahore, Pakistan
[5] Department of Health, Government of Sindh, Pakistan

Correspondence to:
Bilal Iqbal Avan
Associate Professor
Department of Population Health
London School of Hygiene and Tropical Medicine
Keppel St, London WC1E 7HT, United Kingdom
bilal.avan@lshtm.ac.uk

For peer review only

Abstract

Objectives: A dearth of qualitative studies constrains in-depth understanding of health service providers' perspectives and experiences regarding the impact of COVID-19 on their psychological health. This study explored the psychological impact of COVID-19 and psychological needs of public-sector healthcare workers in secondary- and tertiary-level healthcare settings of Pakistan.

Design: An exploratory qualitative study.

Setting: Twenty-five secondary- and eight tertiary-level public hospitals of Sindh and Punjab provinces of Pakistan.

Participants: In-depth interviews were conducted with sixteen health service providers, and forty administrative personnel. Study data were analysed on NVivo 11 using the conventional content analysis technique.

Results: The study identified three overarching themes: (i) psychological impact of COVID-19 on health service providers that included the fear of acquiring infection and transmitting it to their family members, fear of social isolation and stigma, anxiety related to the uncertainty of COVID-19, nervousness due to media exaggeration, and stress associated with excessive workload; (ii) psychological needs of health service providers involved in the COVID-19 crisis and available support from the healthcare system, including the expression of the need for counselling services and safe working conditions, the need for paid rest periods, and the need for appreciation and motivation to work in the pandemic; and (iii) suggestions to address psychological needs of healthcare workers, including provision of specialized mental health care/services, formal training of health managers on managing psychological health needs of health facility staff, and assessment and addressing of the psychological health needs of the health workforce.

Conclusion: The study emphasises the need to strengthen health system preparedness for recognizing and addressing the needs of health professionals. At the system level, there is a need for a specialised unit to provide psychological health services and better communication strategies. At the staff level, continuous motivation and appreciation should be given to healthcare professionals either through monetary incentives or formal acknowledgement of their performance.

Keywords: COVID-19, corona virus, mental health, healthcare workers, psychological

For peer review only

Strengths and limitations of this study

- This study provides an in-depth exploration of experiences of health care professional regarding the psychological impact of COVID-19, as well as their psychological needs and suggestions to address their psychological needs in pandemic crisis.
- Unlike previous urban-focused studies, the participants in this study were invited across twenty-two urban and rural districts in two most populous provinces of Pakistan.
- One key limitation of the study was that the interviews were conducted by phone owing to restricted mobility during the COVID-19 pandemic, which may have introduced some risk of bias as the interviewers did not have the opportunity to build rapport as in face-to-face interviews.

For peer review only

Introduction

Worldwide, health service providers are under insurmountable psychological pressures as they are expected to deal with a number of issues that arise due to the COVID-19 situation[1]. First, due to the increased clinical workload associated with COVID-19 cases, health service providers face increasingly long work hours, often with limited resources and weak infrastructure[2]. Second, they face physical discomfort associated with donning and doffing of personal protective equipment (PPE), or the unavailability of it, which is essential to keep them safe from exposure to the virus[3]. Third, health service providers feel unprepared to deal new and unfamiliar task since the evidence-based clinical treatments for COVID-19 continue to evolve[3] . Finally, there is a very valid fear of autoinoculation and the risk of transmitting the virus to family members[4,5] Unsurprisingly, all these factors take a toll on the mental health of healthcare professionals (HCPs) and result in various psychiatric issues including fear[6], anxiety[1], depression[1], burnout[7], trauma[8], and insomnia[9]. Evidence show that psychological health implications for health workers who are involved in the provision of healthcare during epidemics and pandemics are long-lasting[10,11].

While quantitative studies have significantly outnumbered qualitative investigations[1,3,6,8,12] in terms of assessing the magnitude of mental health problems among HCPs resulting from COVID-19, they are limited in providing an in-depth understanding of HCPs perspectives and experiences regarding the impact of COVID-19 on their mental health. Moreover, most qualitative investigations explored the general experiences of HCPs during COVID-19[13,14], whereas very few have specifically focused on their psychological experiences and they are mainly from high-income countries[12]. A qualitative study from England concluded that COVID-19 patients brought a significant emotional toll, and strained relationships between immediate front-line staff, and their families. At the beginning of the pandemic, staff were driven by adrenalin and optimism; but over time this dissipated to be replaced by exhaustion, numbness, and dreadful expectation of a 'second wave'[15]. Another qualitative study from Pakistan[16] presented a thorough understanding of how the frontline emergency health care workers are dealing with the COVID-19 pandemic, their stress-coping strategies or protective factors, and challenges while dealing with COVID-19 patients. These studies are limited in providing evidence on health systems' preparedness to respond to the mental health needs of HCPs. Earlier evidence during SARS epidemic showed that system-level intervention were effective in reducing health service providers' symptoms of anxiety and depression[1-4,11].

By and large, there is not only dearth of qualitative studies on psychological impact of COVID-19 on health professionals from low- and middle-income countries (LMICs) but there are gaps in existing literature[13]. For example, there is lack information about the psychosocial and mental health needs of health professionals and how well the health system has responded to those needs[12]. Furthermore, these qualitative studies had a predominant focus on hospitals situated in urban settlments or metropolitan cities, while their sample did not have adequate representation from rural settings. Also, the HCPs included in many quanlitative studies were mainly doctors and nurses; few studies have gathered perspectives of other HCPs – such as health administrators[12].

To the best of our knowledge, there is paucity of qualitative evidence to understand the psychological needs of HCPs and the health system response, which is essential to develop context-specific mental health interventions. This study is the first to explore the psychological impact of COVID-19 as well as their psychological needs of health service providers in public healthcare settings of Pakistan from the perspective of health managers and health service providers including doctors and nurses.

For peer review only

Our study aims to answer the following research questions:

- What is the perceived psychological impact of the COVID-19 pandemic on the performance of health service providers in secondary and tertiary care settings in Pakistan?
- What are the health service providers' psychological needs working amid the COVID-19 pandemic, and how well the health system is responding to them?
- What are the recommendations by the health service providers to address their psychological needs?

Methods

Country context

Pakistan has a population of over 225 million inhabitants[17]. The first COVID-19 case was identified in February 2020, and since then a total number of confirmed cases exceed 1.5 million along with 30,574 deaths has been registered (till August 29, 2022)[18]. The country has experienced five waves – each with different variant including omicron, more recently[18]. With a focus on vulnerable groups of HCPs and elderly (65+ years) people, the country embarked on a nation-wide vaccine campaign for COVID-19[18].

Healthcare in Pakistan is delivered through a three-tiered health system. Secondary-level healthcare facilities concerns with provision of technical, therapeutic and diagnostic services. Specialist consultation and hospital admissions fall into this category. The availability of specialised services vary from facility-to-facility across districts dpending upon catchment population and their service needs and demands[19]. It includes Tehsil Head Quarter (THQs) and District Headquarter Hospitals (DHQs). Tertiary healthcare hospitals are for more specialized inpatient care supported by availability of required resources. With few exceptions, these are also affiliated with the medical teaching institutions for graduates and post-graduates[20]. The government of Pakistan has taken several measures to combat this deadly pneumonia virus[21] but it leaves a lot to be desired[22]. The health system of Pakistan is weak[23] and with critical shortage of health workforce, and is not prepared to sustain a major surge in COVID-19 cases[24].

Recent quantitative studies show a considerable impact of COVID-19 on psychological health of healthcare professionals[25,26]. Generally in Pakistan, mental illnesses are stigmatized and widely perceived to have supernatural causes. The psychiatrist to population ratio is about 1 psychiatrist to 0.5–1 million people[27]; this - combined with limited task sharing interventions - resulting a huge treatment gap in the country. The economic burden of mental illnesses in Pakistan as US$ 4.2 billion annually[28].

Design, setting, and participants

Consolidated criteria for reporting qualitative research (COREQ) checklist has been used for designing this qualitative study[29] (see supplementary file 1).

This research employed an exploratory qualitative research design using semi-structured interviews and a criterion purposive sampling approach. Our study was conducted at secondary- and tertiary-level public sector hospitals of two most populous (Sindh and Punjab) provinces of Pakistan. A total of eight teaching hospitals, eight DHQs, and twenty-five THQs were randomly selected from Sindh and Punjab Province. The study was conducted in close collaboration with provincial health departments.

The data collection methods for this research included in-depth interviews (IDIs) with health service providers (doctors, nurses), head of department (HoD), and medical superintendent (MS). In terms of participants' involvement in the care of COVID-19 patients, health managers including MS and HoD was indirectly involved with the management of COVID-19 patients; however, health service providers including doctors and nurses were directly involved in the care of COVID-19 patients. For secondary-level health facilities, we contacted the MS who was invited to participate in the study. Moreover, s/he was further asked to nominate service providers from their respective health facilities who may or may be not involved in the provision of care to COVID-19 patients. Similarly, for teaching hospitals, we contacted the administrative head who identified a focal person on his/her behalf for further coordination. Similar, request was made to the focal person for identification of department heads and service provider. Only 9 participants refused to participate mainly due to their busy schedule and lack of interest.

Eligibility criteria

Health managers and health service providers employed in public sector hospitals of Sindh and Punjab province and dealing directly or indirectly with COVID-19 patients were eligible to participate in the study. Health service providers and hospital management either belonging to private sector hospitals or from provinces other than Sindh and Punjab were excluded. Non-clinical staff such as janitors and technicians were also excluded from the study.

Data collection

Adapting to the COVID-19 situation, a total of 56 (Sindh: 34; Punjab:22) semi-structured telephonic IDIs were conducted individually in the local language (Urdu), by trained female data collectors who had a background in sociology, anthropology and psychology. The interviews are conducted at Aga Khan University Hospital under close supervision of research investigators. Keynotes were also taken by data collectors during the interviews. It was ensure that participant is comfortable during the interview and no one is sitting near to them whose presence could possibly influence his/her responses. To ensure data quality, a few initial interviews were conducted by WH (PhD scholar – health system), AF (PhD scholar – public health) and NA (BS psychology) in presence of training data collectors. The IDIs were conducted until data saturation was achieved [30]. Data collection and data analysis were conducted iteratively to determine the point of data saturation [31,32]. All interviews were completed in the first interaction. Consistent with Creswell and Poth's [31] guidelines on interviewing, all IDIs lasted approximately between 45-60 mins. The interviews were audio-recorded on tablets with consent from study participants, which were later transcribed in the English language by a transcriptionist. The data collection took place between August and October 2020. The transcripts were not returned to the participant due to their busy schedule in the hospitals.

A semi-structured interview guide was designed for IDIs through extensive literature review and consultative approach seeking expert opinions. This guide was developed to interview all research participants. The guide includes five major themes: a) Psychological Impact of COVID-19 on health service providers; b) Psychological needs of health service providers involved in COVID-19 crisis; c) Support mechanisms available to address psychogical needs of health service providers; d) Challenges in addressing psychological needs of health service providers; e) Recommendations to address psychological needs of health service providers. A free flow of information was encouraged, using probes from the participants verbatim to understand their perception of mental health needs during COVID-19.

The interview guide was used flexibly to allow participants to construct their accounts on their own terms. The guide was pilot tested with a non-study sample who share the same characteristics

For peer review only - http://bmjopen.bmj.com/site/about/guidelines.xhtml

as the study sample [31]. The pilot testing provided evidence-based direction to improve the interview guide questions [32] (see supplementary file 2).

Data analysis

All study data including notes, recordings, and transcriptions were uploaded into the NVivo version 11.0 software for data analysis [33]. The analysis looked at convergent and divergent lines of inquiry from the two main study groups, that is, health managers and health service providers (doctors and nurses). All qualitative data including were analysed using conventional content analysis [34,35]. Two independent researchers (AS and NA) read each transcript from beginning to end, and coded text that appeared to describe the psychological impact of COVID-19 as well as the mental health needs of health service providers in public healthcare settings of Pakistan. After open coding of initial few transcripts, independent researchers met to discuss and decide on the preliminary codes (codebook). Researchers then coded the remaining transcripts (and recoded the original ones) using the codebook and added new codes when encountered data that did not fit into an existing code. Once all transcripts had been coded, researchers examined all data within a particular code. Some codes were combined during this process, whereas others were split into subcategories. The discrepancies in coding were resolved through a discussion with a senior researcher. Throughout the analysis, the researcher made reflective notes (memos) to document important learnings from the data [36]. AS and NA independently performed coding to ensure that interpretations are coherent, plausible, and grounded in the study data[32]. Please see supplementary file 3 to see example of content analysis.

Ethical considerations

Ethical approval for this study was obtained from the Aga Khan University Ethical Review Committee (AKU-ERC) – [2020-5186-11602], and National Bioethics Committee of Pakistan – [4-87/COVID-45/NBC/20/393]. The interviews were conducted telephonically. Verbal informed consent was taken from the study participants in which they were informed about the purpose of the study and their rights to participant. The audio recordings were stored in a password-protected folder accessible only to the research team. Permission letters from the Health Departments were issued by the respective provincial governments. These letters along with the consent forms were sent through WhatsApp to the District Health Officers (DHOs) of all the selected districts. Participants were also sent an SMS explaining to them the purpose of the study.

Trustworthiness of the study

Of the five markers suggested by Tracy et al[37] and Lincoln and Guba's[38] to ensure trustworthiness and methodological rigour, we used validity and credibility and reliability/dependability. To ensure credibility, the study triangulated data via two basic types of triangulations: data source triangulation (exploring insights of different groups—hospital managers and health service providers) and investigator triangulation (use of multiple researchers in analysis phase—ASF and NA)[31]. In addition, the primary researcher maintained a reflexive journal during all stages of the research to recognize and acknowledge biases during the research process. For example, reflexivity was used to develop the interview guides, and modify it based on the inputs from other investigators and after the initial interviews, and analyze the data in different groups to tell a story about how the health system responded to deal with the mental health needs of health service professionals during COVID-19

Patient and public involvement

We sought inputs from target audience through pre-testing of interview guides to ensure comprehension of the questions. Furthermore, in consultation with provincial health departments we selected districts where COVID-19 cases were high. The focal person in each selected district was oriented about the study who connected our team with in-charge of health facility to avoid

For peer review only

any miscommunication. Lastly, they also advised about the dissemination of research findings – that is, they specifically emphasis to share it through research briefs to reach wider audience in the country.

Results

A total of 56 semi-structured IDIs were conducted to explore the psychological needs of health service providers amidst COVID-19 in Pakistan. Of 56 IDIs, 16 IDIs were conducted with health service providers, and 40 IDIs were conducted with health managers. All the participants (n = 56) who were approached by the study team agreed to participate. The demographic information for all the study participants is illustrated in Table 1.

Table 1: Characteristics of study participants

Characteristics of study participants		n (%)
Gender (n=56)	Female	9 (16.1)
	Male	47 (83.9)
Designation (n=56)	Hospital Managers	40 (71.4)
	Health Service Providers	16 (28.5)
Facility Type (n=56)	Secondary healthcare hospitals	37 (66.1)
	Tertiary healthcare hospitals	19 (33.9)
Age (n=48)	Mean	46.6
	Median	47
	SD	10.6
Experience (n=53)	Mean	15.4
	Median	15
	SD	9.5

Based on the directed content analysis, three overarching themes were identified: (I) Psychological impact of COVID-19 on health service providers; (II) Psychological needs of health service providers involved in COVID-19 crisis and available support from health system; and (III) Suggestions to address psychological needs of health service providers. These themes and their categories are presented in Table 2.

Table 2: Themes and sub-themes

Themes	Sub-themes
Theme 1: Psychological impact of COVID-19 on healthcare professionals	1.1 Fear of infection, isolation and stigma<br>1.2 Stress due to poor availability of personal protective equipment<br>1.3 Anxiety due to uncertainty of COVID-19<br>1.4 Anxiety and nervousness due to media exaggeration<br>1.5 Excessive workload
Theme 2: Psychological needs of health workforce	2.1 Need to provide counselling services and safe working conditions<br>2.2 Need for paid rest periods, timely payment of workers' salaries, and incentives

For peer review only

involved in COVID-19 crisis and available support from health system	2.3 Need of appreciation and motivation to work in the pandemic
Theme 3: Suggestions to address the psychological needs of healthcare professionals.	3.1 Establish mental health services to address the psychological needs of health workers<br>3.2 Conduct formal training of health managers on managing psychological health needs.<br>3.3 Assess and address psychological needs of the health workforce

1. Psychological impact of COVID-19 on health service providers

1.1 Fear of infection, isolation and stigma

The interviews with hospital managers and health service providers revealed that all health service providers felt fearful of acquiring the COVID-19 infection and transmitting the infection to their family members. A hospital manager mentioned that health service providers are particularly concerned about their parents and children who are vulnerable and might get infected.

"They (HCPs) were afraid for their families that our parents are old and if we carry coronavirus from here then we might not get them infected. Even if we don't show any symptoms, we would get them infected or we would get our children infected. So this fear was also prevailing." (Hospital Manager, Punjab)

Health service providers were fearful that if they did acquire the infection, they would have to isolate themselves from their families. Many workers could not afford to isolate themselves in the event they tested positive for COVID-19.

"They used to say that if the report comes back positive, then how they will stay at home because their homes are small" (Hospital Manager, Sindh)

"Immediately after that, there was fear of isolation, like if you put yourself into isolation who will look after your family and your kids". (Hospital Manager, Punjab)

Both groups of respondents highlighted that few health service providers resigned from their jobs since their families were getting infected because of them. In particular, female health service providers had higher pressure on them from their families to leave their jobs. While some providers refused to work in COVID-19 wards, others threatened health managers to commit suicide, if they were posted there. Health managers verbalized that a lot of the health service providers posted in COVID-19 wards revealed the fear of being isolated and stigmatized.

"Health workers were hospital-bound and were afraid of living alone and being cut off from their families. They also felt the stigma from the people around them". (Hospital Manager, Sindh)

1.2 Stress due to poor availability of personal protective equipment

A major concern among the health service provider was the lack of protective gear available to them. So, treating COVID-19 positive or suspected cases without PPE was escalating their fear of getting infected. They felt that they had been sent to fight a war without weapons.

For peer review only

"Firstly, the PPEs we were using, we had to use them twice or thrice... this was our main issue. We had to wear the mask for two to three days even one week continuously" (Health Service Provider, Punjab)

On the other hand, health managers revealed that some health service providers labelled them as being insensitive and unaware of the problems faced by workers due to lack of PPE availability. However, managers themselves were helpless due to its lack of availability in the market and ended up buying masks and other protective equipment for the staff themselves. But this protective gear was not up to the mark.

"That time if anyone didn't get PPE they (health service providers) would get hyper but we provided PPEs at our facility. We would provide requisite equipment soon so we didn't have to face so much trouble." (Hospital Manager, Punjab)

1.3 Anxiety due to uncertainty of COVID-19

Health service providers reported feeling extremely anxious working with COVID-19 patients. Owing to the novelty and unfamiliarity of the virus along with workers being inadequately equipped with the required knowledge about the disease, they were uncertain about the consequences. Health service providers were also having trouble identifying cases of COVID-19 since there were both, symptomatic and asymptomatic cases. They were not clear about the mode of transmission of the virus.

"One can call it anxiety, you can see it, and they were under a depressive state. All healthcare workers were. From doctors to nursing staff, paramedics, our sweepers, all our workers were going through depression." (Health Service Provider, Sindh)

Some hospital managers had contrasting views about the difficulties faced by their staff while working during the pandemic. They claimed that health service providers were not stressed while working during the COVID-19 pandemic and that none of them came to the managers to express their concerns.

"I don't think their psychological health was affected, I don't think so, and there was stress only in the beginning when they were not aware of the atmosphere" (Hospital manager, Sindh)

"No there was no pressure on them, we were doing many meetings, we were explaining it to them, that there's no issue, this time has come we will solve it Insha'Allah, we will be successful, there wasn't an issue like this in our staff, they were definitely cooperating with us, that's why there was no stress or any problem like that" (Hospital manager, Sindh)

1.4 Anxiety and nervousness due to media exaggeration

Both groups of respondents reported that media has a strong role in mounting anxiety and distress among healthcare workers. The media was seen to exaggerate the magnitude of the situation and there was a bombardment of information. News regarding deaths of workers due to COVID-19 was also creating panic among the majority of the healthcare workers.

"But this novel coronavirus made hype through digital media as no one knew what it was as initially people were stressed" (Hospital Manager, Sindh)

False information was seen to circulate about the negative consequences for people testing positive for COVID-19, which was creating a lot of distress among healthcare workers currently

For peer review only

working in COVID-19 wards. In addition, news related to the long-term complications of COVID-19 increased nervousness among healthcare workers.

"If you take my personal opinion, the hype created regarding this is too much, that has spread too much tension. That has bothered people a lot about what will happen, God forbid if somebody has corona then he will be taken away or this will happen or that will happen, etc. or because of me, my children will suffer". (Health Service Provider, Punjab)

1.5 Excessive workload

Both groups of respondents mentioned that health service providers felt strained and exhausted because of the clinical workload. Due to the shortage of health workforce, the existing pool of health service providers had to bear the burden of extra clinical work. Their duty hours increased along with the number of shifts they had to do per week, which resulted in increased psychological distress among health service providers.

"So far, no one told me that their mental health has been affected. …yes, some people have said that they are depressed…they are feeling tired and have motion sickness due to extra working hours". (Health Manager, Punjab)

The COVID-19 outbreak among health service providers led to further staff shortages and increased mental pressure among health service providers. Both groups of respondents mentioned that even after returning from sick leave, health service providers were not performing at their best. Some of them seen to become weak and some of them would faint. In addition, some workers' tests were negative for the COVID-19 infection, but they still had cough symptoms which would make their co-workers anxious. Hence, they also had to be given leaves.

"As the work load increased, so did the pressure on the HCWs and their efficiency began to decrease". (Health Manager, Sindh)

Both groups of respondents mentioned that their commute time to the hospital also significantly increased due to the unavailability of public transport during the lockdown which further increased the psychological burden among health service providers.

"Our doctors faced a lot of transport issues but we tried our best to support them. Few of our staff were punctual initially, but some of them were not coming at all due to fear of COVID-19. Their wages are less so we reduced the duration of their duties and counselled them a lot after which they started coming to work. They come from far off areas so they should be given space here at the hospital so that they can perform their duties easily". (Health Manager, Sindh)

2. Psychological needs of the health workforce involved in the COVID-19 crisis and available support from health system

2.1 Need to provide counselling services and safe working conditions

Health service providers and health managers highlighted the need for counselling services for health service providers working under pressure to provide care to COVID-19 patients. Health service providers mentioned the desire to receive one-to-one counselling sessions from mental health professionals to reduce the stress and anxiety they were experiencing because of working during COVID-19.

All hospitals should at least provide one-to-one …counselling sessions…to reduce staff anxiety and stress. (Health Service Provider, Sindh, male)

Since no proper counselling services from mental health professionals were available, informal counselling to health service providers was provided by hospital managers. Although the managers were trying to support their staff, they lacked formal training in providing counselling. They motivated them via reinforcing health providers' professional and moral obligations but it did not prove fruitful for most of the workers.

"Whatever knowledge I had regarding the disease and its spread, and I also told them that we must serve humanity, and death is inevitable and I did whatever I could, regarding the moral values, whatever I could make them understand, some of them understood and most of them didn't". (Hospital Manager, Sindh)

"I told them with a passion that the death Allah has written, if we die on this job, they will be martyred" (Hospital Manager, Punjab)

Health managers from few facilities reported that the government had arranged webinars for health service providers to help them manage psychological stress. However, the effectiveness of these training was quite low because many health workers had duties due to which they were unable to attend or sit through them.

In addition, health service providers highlighted the need of working in a safe and secure working environment. They further mentioned that the lack of safety was largely attributed to the inadequate supply of protective equipment. To make up for the lack of PPEs, health service providers resorted to using the same protective gear they had repeatedly for multiple days.

"In many places, we have seen that doctors and paramedical staff were affected and all this is due to the lack of what was needed, for example, the lack of hand sanitizers and masks". (Hospital Manager, Sindh

2.2 Need for paid rest periods, timely payment of workers' salaries, and incentives

Health service providers also highlighted the need for extra rest periods so that they could get enough time to relax from the exhaustive routine and then come back to work. A common strategy employed by the health managers was to give short leaves to the health service providers most affected by the COVID-19 stress. These leaves helped the staff in regaining their mental and physical strength to work.

"They (health service providers) used to say we will do work for 15 days and then require 15 days for holidays, we will relax during that time, I said ok I will give that to you". (Hospital Manager, Sindh)

In addition, the majority of health service providers and health managers reported that lack of additional allowance for staff directly dealing with COVID-19 patients was a demotivating factors especially for paramedical staff.

"The poor paramedical staff who are the first ones to interact with the patients get paid very less. The government did not do anything for them because the staff used to complain that we are

getting the same salaries while having maximum exposure to COVID-19." (Health Service Provider, Punjab)

Health service providers also wanted to be given monetary incentives because working during the pandemic meant that their lives were at stake. This would also help to increase their motivation to work during such difficult conditions.

"They should be given incentive because they are keeping their life on risk" (Health Service Provider, Sindh)

2.2 2.3 Need for appreciation and motivation to work in the pandemic

Hospital managers and health service providers reported that workers needed appreciation for all the hard work they were doing. And providing appreciation would, in turn, lead to the workers becoming motivated to continue their efforts. Health service providers were putting their lives at stake while working in such difficult conditions without adequate sources, hence their efforts should be commended.

"Then they (health managers) should arrange programs in which they appreciate and motivate their employees and provide them proper guidance then it will have major positive effects on mental health". (Health Service Provider, Punjab)

It was also suggested that the staff should be given certificates to appreciate the work they were doing and to hold regular meetings with them to see work progress and understand their needs. Health service providers mentioned that motivating sessions are also needed to boost morale of the workers who are working in such difficult conditions.

"I told my hospital director that we need a psychologist. I also highlighted that our human resource team should be given appreciation certificates other than monetary incentives which might boost their motivation to work. Meetings should also be conducted with them to ask them about their work and what else they need since this is also lacking currently". (Hospital Manager, Sindh)

3) Suggestions to address psychological needs of healthcare professionals

3.1 Establish mental health services to address the psychological needs of health workers

Both groups of respondents stressed that there needed to be an increase in the budget allocated to the health sector to establish mental health services for health service providers working during COVID-19. More funds and resources would lead to betterment for healthcare workers. Hiring mental health professionals including psychologists and psychiatrists to support the health service providers' psychologically were strongly recommended and emphasized repeatedly by health managers.

With regards to the roles of provincial governments in improving the psychological health of healthcare professionals, there was a need for the implementation of formal programs addressing psychological health. The Government was also advised to arrange seminars on psychological health for health service providers alongside launching a mobile application for providing relevant information on the topic.

"There should be seminars and sessions and the Punjab government should launch an app regarding this so that we get information from it regarding mental and physical health and keep on updating it. This will improve the situation". (Health Service Provider, Punjab)

For peer review only

Hospital managers were also of the view that government officials should visit hospitals and have discussions with all levels of health service providers to understand their experiences and needs. This would enable the government to formulate effective strategies in dealing with problems faced by health service providers. Some health service providers complained that their health managers were not bothered if their staff was facing any issues.

"Nobody bothers to discuss any issue faced by workers. They only give orders to perform their duties. Our hospital managers, they don't have any concerns if workers are having any problems or not" (Health Service Provider, Sindh)

Sorting logistical issues such as providing transportation facilities for health service providers to and from quarantine centres was also stressed upon.

3.2 Conduct formal training of health managers on Managing Mental Health needs

Since hospital managers were directly overseeing the health service providers and had the most interaction with them, their formal training in providing basic counselling to the staff was deemed necessary. Health service providers wanted managers to hold daily meetings with them where they could discuss the difficulties they were facing while caring for COVID-19 positive patients.

"The administrator of the hospital should talk about it from every doctor. He should do meeting with them to see if they face any mental pressure or not and if he has so the problem then he should sort out his problem" (Health Service Provider, Sindh)

Some health service providers also remarked that hospital managers should be more approachable so that the staff feels comfortable in sharing their concerns with them.

"If employees are guided properly in monthly or weekly meetings, their issues are being heard whatever they are. There should be less difference of job between an employee and MS or head of the department so that he can go and discuss his problems with him, so many of problems can be solved" (Health Service Provider, Punjab)

3.3 Assess and address mental health needs of the health workforce

Another recommendation for the future was for the government and health managers to plan all the measures the health system would need to take in the event of such a crisis. They should have observed the situation in countries such as China where the outbreak first began and started thinking of strategies to get a better grip of the impending problems.

"We don't know if this pandemic will subside or not. God Forbid if this second waves comes and traffic of patients increases, then what will we do? This way we will get depressed, so proper training should definitely be given, mental health workers should come, go to the institution and guide the HCWs." (Health Manager, Sindh)

Health service providers also suggested that keeping in mind the difficult conditions they were eventually going to have to work in, there should have been sessions that would mentally prepare them for the latter.

"When you send a doctor in the COVID-19 ward, there should have been a week-long training with a psychologist, to explain them the situation, what will be their duties, the government has

For peer review only

selected you, for this task, they should have been prepared and there is nothing to worry about, the mortality rate is low, and most people recover" (Health Service Provider, Sindh)

Discussion

The large-scale unprecedented public health catastrophe in the form of COVID-19, placed the frontline HCPs, who are directly involved with all aspects of care of COVID-19 patients, at risk of developing psychological distress and other mental health symptoms.[39] Our study provided an in-depth investigation into the psychological impact of COVID-19 on HCPs and mental health needs of different cadres (health service providers and administrators) in public health sector settings.

We documented an adverse perceived psychological impact experienced by health service providers while working during the pandemic as revealed in other studies from other countries[12,40]. The health service providers expressed several explanations for psychological stress including the fear of acquiring infection and transmitting it to their family members, fear of social isolation and stigma, anxiety related to the uncertainty of COVID-19, nervousness due to media exaggeration, and stress associated with excessive workload. Evidence that show that exaggeration by media tend to negatively affect the psychological health of HCPs[41,42]. Similarly, lack of knowledge about COVID-19 infection is likely to increase anxiety/depression among physicians[43] and inadequate precautionary measures in the workplace causes fear and anxiety among HCPs[44]. Furthermore, overburdening due to high intensity of care and increased volume of clinical services have been associated with work-related stress, burn out, and post-traumatic stress disorder, which in turn negatively affect job performance and quality of services[4542].

Perceptions of the two groups of respondents (service providers and health managers) on most issues were largely similar. However, at some instances, we noted divergent views among the two groups regarding the impact of COVID-19 on the psychological health of health service providers. It is worrisome to note that while health service providers claimed to experience significant adverse psychological effects due to COVID-19, some health managers denied that their staff faced any stress or difficulty working during the pandemic. These contradictory views may be attributed to a disconnect or lack of communication between service providers and health managers. Another reason for this denial from health managers could be an attempt to express that things are managed well by them since they are generally responsible for the safety and well-being of service providers. Nonetheless, it is important to investigate the causes of such contrasting views about the same phenomenon by different groups, especially when it affects the health system at large.

The study revealed the psychological health needs of health service providers including the need to provide counselling services and safe working conditions, the need for paid rest periods and timely payment of workers' salaries, and the need for appreciation and motivation to work in the pandemic. This finding is consistent with other studies where HCWs expressed similar needs [46,47]. For example, previous experience from the outbreak of Ebola in western Africa highlighted the need to provide risk allowance for motivating and retaining healthcare workers[7]. Our study also highlighted the need to provide risk allowance to health service providers. In addition, our study emphasized the need to provide timely salaries to the health service providers, and extra rest periods. Both groups of respondents revealed that the health system response in the given scenario has been weak given the lack of existing support mechanisms to address the mental health needs of the health workforce during COVID-19. Inefficient management of the pandemic from healthcare system was also reported in countries[40,48,49].

For peer review only - http://bmjopen.bmj.com/site/about/guidelines.xhtml

Finally, the health service providers and health managers provided three suggestions to address the mental health needs of health service providers in the future including the provision of mental health services to address the psychological needs of health service providers, conducting formal training of health managers on managing and anticipating the psychological health needs of the health workforce to guide future initiatives. Experiences from similar outbreaks suggest that early psychological intervention and the establishment of early support systems are particularly important to promote the emotional release and improve health service providers' well-being [50]. Similar interventions have been used in past to address psychological health issues in healthcare workers during infection disease outbreak[51]. Capacity building of health facility staff on the provision of psychosocial support is also recommended in the World Health Organization (2020) interim guidance document on COVID-19[42]. Call centres have been established in many countries – including Pakistan[52] - to provide needs-based psychological support to HCPs. In Pakistan, the “WeCare” programme probably needs to expand its scale of services to effectively address the psychosocial and mental health needs health care professional. Our study will use knowledge translation tools to share the key findings of this qualitative study with a diverse audience including policymakers, knowledge users, clinicians, researchers, health service providers, and hospital managers. Concerning future research direction, insights from the qualitative study would help in streamlining health system response in addressing the mental needs of the health workforce working in COVID-19 pandemic.

Strengths and limitations

The study has several strengths and limitations. First, the study used multiple cadres of the health system (health service providers and health managers) which allowed researchers to look for converging and diverging lines of inquiry to identify common themes/ concepts and incongruence between data sources. Second, our study sample provides adequate representation from rural settings in the country. Third, the study was conducted in close collaboration with the provincial governments.However, this study is also subject to limitations. First, the study was conducted with a focus on public sector hospitals in only two provinces of Pakistan (Sindh and Punjab), which would limit the transferability of the findings to other provinces [53]. Guba and Lincoln argue for the concept of ‘fittingness’, which emphasizes analysing the degree to which situation studied matches other situation in which one is interested, provides a more workable way of thinking about transferability[53]. Thus, this study may provide insights for similar public hospitals across Pakistan that are interested in understanding the psychological impact and mental health needs of health service providers during COVID-19. Second, the study was unable to carry out member checking with study participants, which may have affected the validity of study findings. Lastly, the authors did not have the opportunity to build rapport and obtain no-verbal cues during interviews since the participants were interviewed telephonically due to the COVID-19 situation, In addition, the interviewees might have been distracted during interviews by the activities in their environment and might have experienced fatigue due to long telephonic interviews

Conclusion

Our findings show that the health system of Pakistan was not prepared to deal with the psychological needs of health service professionals during COVID-19. This information is crucial in anticipating and preparing for tackling possible future pandemics. Health managers and health service providers shared similar concerns about working during the outbreak. Multiple recommendations are made to the government stakeholders for initiating nationwide initiatives for catering to the psychological needs of healthcare professionals. At the system: a) communication strategies should be devised to ensure better communication between health managers and health service providers at all hierarchies so that the needs and concerns timely are heard and taken care of; and b) there is a need to establish a specialised unit that should provide psychological health services to mitigate the detrimental effects of COVID-19 on the psychological

For peer review only

health of healthcare professionals. At the staff level: healthcare professionals should be given recognition to higher their morale either through monetary incentives or through formal appreciation based on their performance.

Declarations

Ethical considerations

Ethical approval for this study was obtained from the Aga Khan University Ethical Review Committee (AKU-ERC) – [2020-5186-11602], and National Bioethics Committee of Pakistan – [4-87/COVID-45/NBC/20/393]. Verbal informed consent was taken from all study participants.

Competing interests

The authors have declared that no competing interests exist.

Funding

This work was co-funded by the Aga Khan University, Pakistan (ID: 20051) and the World Health Organization (ID: 202568710-1)

Contributors

WH and BIA conceptualised the study with inputs from BK, ZF and SS. WH, BIA, BK, ZF, and AF developed interview guide. HJ and MW facilitated in conducting the study. AF and NA performed data analysis, supported by WH. WH and AF took the lead in writing the manuscript. All authors critically reviewed the manuscript and provided feedback in shaping the write-up, analysis and presentation of results. WH addressed comments from co-authors and finalized the manuscript.

Disclaimer

The views expressed in this manuscript are those of the authors and contributors, and do not necessarily reflect those of the World Health Organization, or the organization to which the authors are affiliated.

Acknowledgements

We are grateful to our implementing partners: Department of Health, Government of Sindh; Primary and Secondary Healthcare Department (P&SHD) and Specialized Healthcare & Medical Education Department from Government of Punjab for extending their full their support and guidance to conduct this survey. We are also thankful to all our study participants for sparing their precious time and made themselves available for the interviews. Last but not the least, we thank our data collectors and project management team: Zafar Dehraj, Imran Sheikh, Sajid Brohi and Ghani Muhammad who managed the implementation of this study in a proficient manner during the pandemic.

Data availability statement

No additional data available.

For peer review only - http://bmjopen.bmj.com/site/about/guidelines.xhtml

References

1. Li Y, Scherer N, Felix L*, et al.* Prevalence of depression, anxiety and post-traumatic stress disorder in health care workers during the COVID-19 pandemic: A systematic review and meta-analysis. *PLoS ONE* 2021; 16:e0246454.https://doi.org/10.1371/journal.pone.0246454

2. Shigemura J, Ursano RJ, Morganstein JC*, et al.* Public responses to the novel 2019 coronavirus (2019-nCoV) in Japan: Mental health consequences and target populations. *Psychiatry Clin Neurosci* 2020; 2020/02/23:281-282.https://pubmed.ncbi.nlm.nih.gov/32034840

3. Huang JZ, Han MF, Luo TD*, et al.* Mental health survey of medical staff in a tertiary infectious disease hospital for COVID-19. *Zhonghua Lao Dong Wei Sheng Zhi Ye Bing Za Zhi* 2020; 38:192-195.https://doi.org/10.3760/cma.j.cn121094-20200219-00063

4. Xiang YT, Yang Y, Li W*, et al.* Timely mental health care for the 2019 novel coronavirus outbreak is urgently needed. *Lancet Psychiatry* 2020; 2020/02/04:228-229.https://pubmed.ncbi.nlm.nih.gov/32032543

5. Kang L, Li Y, Hu S*, et al.* The mental health of medical workers in Wuhan, China dealing with the 2019 novel coronavirus. *The Lancet Psychiatry* 2020; 7:e14.http://www.sciencedirect.com/science/article/pii/S221503662030047X

6. Hu D, Kong Y, Li W*, et al.* Frontline nurses' burnout, anxiety, depression, and fear statuses and their associated factors during the COVID-19 outbreak in Wuhan, China: A large-scale cross-sectional study. *EClinicalMedicine* 2020; 24:100424.https://www.sciencedirect.com/science/article/pii/S2589537020301681

7. Khasne RW, Dhakulkar BS, Mahajan HC*, et al.* Burnout among Healthcare Workers during COVID-19 Pandemic in India: Results of a Questionnaire-based Survey. *Indian J Crit Care Med* 2020; 24:664-671.https://pubmed.ncbi.nlm.nih.gov/33024372

8. Khattak SR, Saeed I, Rehman SU*, et al.* Impact of Fear of COVID-19 Pandemic on the Mental Health of Nurses in Pakistan. *Journal of Loss and Trauma* 2021; 26:421-435.https://doi.org/10.1080/15325024.2020.1814580

9. Raza A, Matloob S, Abdul Rahim NF*, et al.* Factors Impeding Health-Care Professionals to Effectively Treat Coronavirus Disease 2019 Patients in Pakistan: A Qualitative Investigation. *Front Psychol* 2020; 11:572450.https://pubmed.ncbi.nlm.nih.gov/33240162

10. Lee SM, Kang WS, Cho AR*, et al.* Psychological impact of the 2015 MERS outbreak on hospital workers and quarantined hemodialysis patients. *Compr Psychiatry* 2018; 2018/10/13:123-127.https://pubmed.ncbi.nlm.nih.gov/30343247

11. Li Z, Ge J, Yang M*, et al.* Vicarious traumatization in the general public, members, and non-members of medical teams aiding in COVID-19 control. *Brain Behav Immun* 2020; 2020/03/10:916-919.https://pubmed.ncbi.nlm.nih.gov/32169498

For peer review only

12. Huerta-Gonzalez S, Selva-Medrano D, Lopez-Espuela F, *et al.* The Psychological Impact of COVID-19 on Front Line Nurses: A Synthesis of Qualitative Evidence. *Int J Environ Res Public Health* 2021; 18.

13. Chemali S, Mari-Saez A, El Bcheraoui C, *et al.* Health care workers' experiences during the COVID-19 pandemic: a scoping review. *Human Resources for Health* 2022; 20:27.https://doi.org/10.1186/s12960-022-00724-1

14. Billings J, Ching BCF, Gkofa V, *et al.* Experiences of frontline healthcare workers and their views about support during COVID-19 and previous pandemics: a systematic review and qualitative meta-synthesis. *BMC Health Services Research* 2021; 21:923.https://doi.org/10.1186/s12913-021-06917-z

15. Bennett P, Noble S, Johnston S, *et al.* COVID-19 confessions: a qualitative exploration of healthcare workers experiences of working with COVID-19. *BMJ Open* 2020; 10:e043949.http://bmjopen.bmj.com/content/10/12/e043949.abstract

16. Munawar K, Choudhry FR. Exploring stress coping strategies of frontline emergency health workers dealing Covid-19 in Pakistan: A qualitative inquiry. *Am J Infect Control* 2021; 49:286-292.https://www.sciencedirect.com/science/article/pii/S0196655320306386

17. Population Reference Bureau. 2021 World Population Data Sheet. 2021. Washington DC, Population Reference Bureau. 28-9-2021.
Ref Type: Online Source

18. National Information Technology Board. COVID-19 Dashboard. 27-4-2020. Islamabad, Government of Pakistan. 27-4-2020.
Ref Type: Online Source

19. Thavar I, Khalid M. Secondary level minimum health services delivery package for secondary care hospitals (MHSDP). 2-11-2016. Islamabad, Ministry of National Health Services Regulations & Coordination, Government of Pakistan. 30-8-2022.
Ref Type: Report

20. Hassan A, Mahmood K, Bukhsh HA. Healthcare System Of Pakistan. *International Journal of Advanced Research and Publication* 2017; 1:170-173.https://www.ijarp.org/published-research-papers/oct2017/Healthcare-System-Of-Pakistan.pdf

21. Waris A, Atta UK, Ali M, *et al.* COVID-19 outbreak: current scenario of Pakistan. *New Microbes and New Infections* 2020; 35:100681.http://www.sciencedirect.com/science/article/pii/S2052297520300330

22. Khalid A, Ali S. COVID-19 and its Challenges for the Healthcare System in Pakistan. *Asian Bioethics Review* 2020; 12:551-564.https://dx.doi.org/10.1007%2Fs41649-020-00139-x

23. Fullman N, Yearwood J, Abay SM, *et al.* Measuring performance on the Healthcare Access and Quality Index for 195 countries and territories and selected subnational locations: a systematic analysis from the Global Burden of Disease Study 2016. *Lancet* 2018; 391:2236-2271.https://doi.org/10.1016/S0140-6736(18)30994-2

For peer review only

24. Abdullah MA, Mukhtar F, Wazir S, *et al.* The Health Workforce Crisis in Pakistan: A Critical Review and the Way Forward. *World Health & Population* 2014; 15:4-12.https://www.longwoods.com/product/24031

25. Riaz B, Rafai WA, Ussaid A, *et al.* The psychological impact of COVID-19 on healthcare workers in Pakistan. *Future Healthc J* 2021; 8:e293.http://www.rcpjournals.org/content/8/2/e293.abstract

26. Ullah I, Khan KS, Ali I, *et al.* Depression and anxiety among Pakistani healthcare workers amid COVID-19 pandemic: A qualitative study. *Annals of Medicine and Surgery* 2022; 78:103863.https://www.sciencedirect.com/science/article/pii/S2049080122006239

27. Karim S, Saeed K, Rana MH, *et al.* Pakistan mental health country profile. *International Review of Psychiatry* 2004; 16:83-92.https://doi.org/10.1080/09540260310001635131

28. Malik MA, Khan MM. Economic Burden of Mental Illnesses in Pakistan. *The Journal of Mental Health Policy and Economics* 2016; 19:155-166.https://pubmed.ncbi.nlm.nih.gov/27572143/#:~:text=The%20estimated%20average%20yearly%20cost,million%20to%20USD%204007.01%20million).

29. Tong A, Sainsbury P, Craig J. Consolidated criteria for reporting qualitative research (COREQ): a 32-item checklist for interviews and focus groups. *Int J Qual Health Care* 2007; 19:349-357.https://doi.org/10.1093/intqhc/mzm042

30. Fusch PI, Ness LR. Are We There Yet? Data Satur et? Data Saturation in Qualitative Research. *The Qualitative Report* 2015; 20:1408-1416.https://nsuworks.nova.edu/cgi/viewcontent.cgi?article=2281&context=tqr

31. Creswell JW, Poth CN. Qualitative Inquiry and Research Design: Choosing Among Five Approaches. Fourth Edition ed. SAGE Publishing; 2017.

32. Yin RK. Case Study Research and Applications: Design and Methods. Sixth Edition ed. SAGE Publishing; 2017.

33. NVivo qualitative data analysis software [QSR International Pty Ltd.; 2015.

34. Hsieh HF, Shannon SE. Three Approaches to Qualitative Content Analysis. *Qual Health Res* 2005; 15:1277-1288.https://doi.org/10.1177/1049732305276687

35. Erlingsson C, Brysiewicz P. A hands-on guide to doing content analysis. *African Journal of Emergency Medicine* 2017; 7:93-99.https://www.sciencedirect.com/science/article/pii/S2211419X17300423

36. Birks M, Chapman Y, Francis K. Memoing in qualitative research: Probing data and processes. *Journal of Research in Nursing* 2008; 13:68-75.https://doi.org/10.1177/1744987107081254

37. Tracy SJ. Qualitative Quality: Eight ΓÇ£Big-TentΓÇ¥ Criteria for Excellent Qualitative Research. *Qualitative Inquiry* 2010; 16:837-851.https://doi.org/10.1177/1077800410383121

38. Lincoln YS, Guba EG. Naturalistic Inquiry. SAGE Publications, Inc; 1985.

For peer review only

39. Wu P, Fang Y, Guan Z*, et al.* The Psychological Impact of the SARS Epidemic on Hospital Employees in China: Exposure, Risk Perception, and Altruistic Acceptance of Risk. *Can J Psychiatry* 2009; 54:302-311.https://doi.org/10.1177/070674370905400504

40. Mohammadi F, Farjam M, Gholampour Y*, et al.* Caregivers' perception of the caring challenges in coronavirus crisis (COVID-19): a qualitative study. *BMC Nursing* 2021; 20:102.https://doi.org/10.1186/s12912-021-00607-1

41. Su Z, McDonnell D, Wen J*, et al.* Mental health consequences of COVID-19 media coverage: the need for effective crisis communication practices. *Globalization and Health* 2021; 17:4.https://doi.org/10.1186/s12992-020-00654-4

42. World Health Organization. Health workforce policy and management in the context of the COVID-19 pandemic response: interim guidance, 3 December 2020. 3-12-2020. Geneva, World Health Organization. 10-10-2021.

Ref Type: Report

43. Amin F, Sharif S, Saeed R*, et al.* COVID-19 pandemic- knowledge, perception, anxiety and depression among frontline doctors of Pakistan. *BMC Psychiatry* 2020; 20:459.https://doi.org/10.1186/s12888-020-02864-x

44. Hoernke K, Djellouli N, Andrews L*, et al.* Frontline healthcare workers' experiences with personal protective equipment during the COVID-19 pandemic in the UK: a rapid qualitative appraisal. *BMJ Open* 2021; 11:e046199.http://bmjopen.bmj.com/content/11/1/e046199.abstract

45. Shaikh S, Baig LA, Hashmi I*, et al.* The magnitude and determinants of violence against healthcare workers in Pakistan. *BMJ Global Health* 2020; 5:e002112.http://gh.bmj.com/content/5/4/e002112.abstract

46. Martins RS, Bhatti OA, Mian AI. Violence Against Health Care Workers in Pakistan During the COVID-19 Pandemic. *JAMA Health Forum* 2020; 1:e201263.https://doi.org/10.1001/jamahealthforum.2020.1263

47. Strengthening the frontline: How primary health care helpshealth systems adapt during the COVID-19 pandemic. 10-2-2021. OECD Policy Responses to Coronavirus (COVID-19). 12-5-2021.

Ref Type: Report

48. Kalateh Sadati A, Zarei L, Shahabi S*, et al.* Nursing experiences of COVID-19 outbreak in Iran: A qualitative study. *Nurs Open* 2021; 8:72-79.https://doi.org/10.1002/nop2.604

49. Bijani M, Karimi S, Khaleghi A*, et al.* Exploring senior managers perceptions of the COVID-19 Crisis in Iran: a qualitative content analysis study. *BMC Health Services Research* 2021; 21:1071.https://doi.org/10.1186/s12913-021-07108-6

50. Sun N, Wei L, Shi S*, et al.* A qualitative study on the psychological experience of caregivers of COVID-19 patients. *Am J Infect Control* 2020; 2020/04/08:592-598.https://pubmed.ncbi.nlm.nih.gov/32334904

For peer review only

51. Zace D, Hoxhaj I, Orfino A, *et al.* Interventions to address mental health issues in healthcare workers during infectious disease outbreaks: A systematic review. *J Psychiatr Res* 2021; 136:319-333.https://www.sciencedirect.com/science/article/pii/S0022395621000868

52. Ministry of National Health Services Regulation and Coordination (MoNHSRC). We CARE. 2020. Islamabad, Government of Pakistan. 18-9-2021.
Ref Type: Online Source

53. Donmoyer R. Generalizability and the Single-Case Study. In: Gomm R, Hammersley M, Foster P, editors. Case Study Method. SAGE publications Ltd.; 2009.

For peer review only

COREQ (COnsolidated criteria for REporting Qualitative research) Checklist

A checklist of items that should be included in reports of qualitative research. You must report the page number in your manuscript where you consider each of the items listed in this checklist. If you have not included this information, either revise your manuscript accordingly before submitting or note N/A.

Topic	Item No.	Guide Questions/Description	Reported on Page No.
**Domain 1: Research team and reflexivity**			
Personal characteristics			
Interviewer/facilitator	1	Which author/s conducted the interview or focus group?	6
Credentials	2	What were the researcher's credentials? E.g. PhD, MD	6
Occupation	3	What was their occupation at the time of the study?	6
Gender	4	Was the researcher male or female?	6
Experience and training	5	What experience or training did the researcher have?	6
Relationship with participants			
Relationship established	6	Was a relationship established prior to study commencement?	6
Participant knowledge of the interviewer	7	What did the participants know about the researcher? e.g. personal goals, reasons for doing the research	7
Interviewer characteristics	8	What characteristics were reported about the inter viewer/facilitator? e.g. Bias, assumptions, reasons and interests in the research topic	N/A
**Domain 2: Study design**			
Theoretical framework			
Methodological orientation and Theory	9	What methodological orientation was stated to underpin the study? e.g. grounded theory, discourse analysis, ethnography, phenomenology, content analysis	5
Participant selection			
Sampling	10	How were participants selected? e.g. purposive, convenience, consecutive, snowball	5
Method of approach	11	How were participants approached? e.g. face-to-face, telephone, mail, email	6
Sample size	12	How many participants were in the study?	6
Non-participation	13	How many people refused to participate or dropped out? Reasons?	5
Setting			
Setting of data collection	14	Where was the data collected? e.g. home, clinic, workplace	6
Presence of nonparticipants	15	Was anyone else present besides the participants and researchers?	6
Description of sample	16	What are the important characteristics of the sample? e.g. demographic data, date	8
Data collection			
Interview guide	17	Were questions, prompts, guides provided by the authors? Was it pilot tested?	6
Repeat interviews	18	Were repeat inter views carried out? If yes, how many?	6
Audio/visual recording	19	Did the research use audio or visual recording to collect the data?	6
Field notes	20	Were field notes made during and/or after the interview or focus group?	6
Duration	21	What was the duration of the inter views or focus group?	6
Data saturation	22	Was data saturation discussed?	6

For peer review only

Transcripts returned	23	Were transcripts returned to participants for comment and/or correction?	6
**Domain 3: analysis and findings**			
Data analysis			
Number of data coders	24	How many data coders coded the data?	6
Description of the coding tree	25	Did authors provide a description of the coding tree?	6
Derivation of themes	26	Were themes identified in advance or derived from the data?	6
Software	27	What software, if applicable, was used to manage the data?	6
Participant checking	28	Did participants provide feedback on the findings?	6
Reporting			
Quotations presented	29	Were participant quotations presented to illustrate the themes/findings? Was each quotation identified? e.g. participant number	7
Data and findings consistent	30	Was there consistency between the data presented and the findings?	7
Clarity of major themes	31	Were major themes clearly presented in the findings?	9
Clarity of minor themes	32	Is there a description of diverse cases or discussion of minor themes?	11

Developed from: Tong A, Sainsbury P, Craig J. Consolidated criteria for reporting qualitative research (COREQ): a 32-item checklist for interviews and focus groups. International Journal for Quality in Health Care. 2007. Volume 19, Number 6: pp. 349 – 357

Once you have completed this checklist, please save a copy and upload it as part of your submission. DO NOT include this checklist as part of the main manuscript document. It must be uploaded as a separate file.

For peer review only

COVID-19: psychosocial and mental health needs of health workers during the outbreak management in Pakistan

IN-DEPTH INTERVIEW GUIDE FOR HEALTH MANAGERS AND SERVICE PROVIDERS

S.no	Questions	Responses	Skip
**SECTION 1: PARTICIPANT CONTACT SHEET**			
Please complete before the interview.			
101	Name of province	Sindh 1<br>Punjab 2	
102	Name of district		
103	Name of TALUKA / Tehsil		
104	Name of Union Council		
105	Type of health facility	Basic Health Unit (BHU) 1<br>Rural Health Centre (RHC) 2<br>Taluka Head Quarter (THQ) 3<br>District Head Quarter (DHQ) 4<br>Tertiary care hospital 5<br>Specialised Isolation Centre 6	
106	Name of health facility / Hospital		
107	Date of interview	______ / _______ / _____________<br>dd mm yyyy	
**SECTION 2: PARTICIPANTS PROFILE**			
201	Sex	Male 1<br>Female 2	
202	Marital status	Never married 1<br>Currently married 2<br>Divorced 3<br>Separated 4<br>Widowed 5	
203	Age (in years)	Years [ ][ ]	
204	Number of living children	Children [ ][ ]	
205	Do you live in a nuclear family or joint family?	Nuclear 1<br>Joint 2	
206	Highest level of education completed?	Matriculation 1<br>Intermediate 2<br>Bachelors 3<br>Masters / M.Phil. 4<br>PhD 5	
207	Professional qualification	Lady Health Visitor 1<br>Lady Health Worker 2	

Version 1.0 Last updated 29th June 2020

For peer review only

For peer review only - http://bmjopen.bmj.com/site/about/guidelines.xhtml

		Midwife 3<br>Nurse 4<br>M.B.B.S 5	
208	What is the current position you hold in the organization?	Lady health supervisors (LHSs) 1<br>Managers from secondary level health facility (DHQ/THQ) 2<br>Medical superintendent of tertiary care teaching hospital 3<br>Specialist from Field isolation center, Karachi 4<br>Others specify______________________ 5	
209	How long you have been working in this health facility / catchment area?	Years [ ][ ]	
210	What is the total years of your professional experience?	Years [ ][ ]	

For peer review only

For peer review only - http://bmjopen.bmj.com/site/about/guidelines.xhtml

No.	Questions	Responses	Probe
**1**	**THEME : Understanding psychological impact of covid-19 on healthcare workers**		
	**First, I would like to ask you some questions to understand the psychological impact of covid-19 on healthcare workers**<br>**The psychological adverse outcomes may include stress, anxiety, depression, burnout, post-traumatic stress disorder, fear-triggered panic attacks, psychosomatic symptoms, psychological violence, fatigue, stigma etc.**		
1.1	How healthcare workers feel about working under tremendous pressure with COVID -19 situation?		Probes:<br>• Can you use example to explain the situation?
1.2	In your opinion, how has COVID-19 affected mental health of healthcare workers?		Probes:<br>• How healthcare workers are dealing with anxiety and fear related to managing COVID-19 inpatients?<br>• Is it affecting healthcare workers both negatively and positively?
1.3	Can you specify the reasons for adverse psychological outcomes among healthcare workers?		Probes:<br>• Reasons related to procedural changes (increased workload, long working hours)
1.4	How negative emotions among healthcare workers will affect the overall functioning of organisations/ institutions?		Probes:<br>• Do you think negative emotions among health worker may lead to poor health worker attention, understanding and decision-making ability
1.5	Do you think health workers have capacity to cope with psychological risk?		Probes:<br>• If yes, what are the coping mechanisms of healthcare workers?<br>• If no, why do you think healthcare workers lack capacity to cope with psychological risk?
1.6	Do you think psychological needs of population has changed in recent times?		Probes:<br>• How health system is responding to those psychological needs?<br>• Are health workers prepared to address changing psychological needs of patients with covid-19?
**2**	**THEME: Identifying psychological needs of health workforce involved in covid-19 crisis**		
	**Now I would like to ask some questions to understand the psychological needs of health workforce involved in covid-19 crisis. The psychological needs of healthcare workers may include enabling working environment, safe and secured hospital environment, adequate staffing, support from mentors and floor managers, support from mental health counsellors, etc.**		
2.1	What are the psychological needs of health workforce involved in covid-19 crisis		Probes:<br>• How did you get to know about their psychological needs?

For peer review only - http://bmjopen.bmj.com/site/about/guidelines.xhtml

2.2	Do you think healthcare workers are able to talk about their psychological needs and concerns?		Probes:<br>• If yes, please share examples of some instances where healthcare workers have shared their psychological needs<br>• If no, why healthcare workers are not able to talk about their concerns?
2.3	As a ______, how do you see your role in identifying the psychological needs of the health workforce		
**3**	**THEME: Support mechanisms available to address mental health needs of healthcare workers**		
	I would like to know the support mechanisms/ processes available to address mental health needs of healthcare workers. These support mechanisms may include providing mental health support services, delivering counselling sessions, supporting workers through mental health helpline, etc.		
3.1	What are the mechanisms available to support the mental health needs of healthcare workers?		Probes:<br>• What interventions are currently used to support the mental health needs of healthcare workers?<br>•
3.2	Do you think healthcare workers are able to receive adequate mental health support with these processes?		Probes:<br>• Are they effective? Are these interventions appreciated by current health workforce?
3.3	Do you think your institution/health facility is prepared to manage mental health needs of the healthcare workers?		Probes:<br>• If yes, please share a recent experience whereby mental health needs of a health worker were adequately addressed.<br>• Do you think health system is currently prepared to address the mental health needs of health care workers<br>○ If yes, why? If no, why?
3.4	Have you received any formal training/ session on supporting mental health needs of the healthcare workers?		Probes:<br>• How many times the training was conducted and for whom?<br>• Can you tell me the objective of each training?<br>• What aspects were covered in the training?<br>• Who conducted those trainings?<br>• What was the duration of each training?
3.5	As a ______, how do you see your role in addressing the psychological needs of the health workforce		Probes:<br>• How do you provide the support to your staff?<br>• Do you feel confident while addressing the mental health needs of your staff?

For peer review only - http://bmjopen.bmj.com/site/about/guidelines.xhtml

			• How do you feel about your capacity in terms of addressing psychological needs of your staff<br>• How effective it is?
4	**Theme: Understanding challenges in addressing mental health needs of healthcare workers**		
	Now I would like to understand the challenges you face while addressing mental health needs of health care workers like lack of adequate trainings, tools and resources		
4.1	What are some of the barriers you face in addressing mental health needs of healthcare workers?		Probes:<br>• Do you think lack of trainings/resources as one of the barriers to addressing mental health needs of healthcare workers?<br>• How health system can address these barriers?
4.2	In your opinion, how these barriers could be handled efficiently, to ensure adequate mental health support for healthcare workers?		
5	**Theme: Suggestions and recommendations to address psychological needs of health care workers**		
	Here I would like to understand how institutions could provide mental health support to healthcare workers. Specifically, we would like to understand the interventions that the health system can offer to alleviate psychological burden of healthcare workers.		
5.1	What kind of relief healthcare workers expect to manage with these pressures?		
5.2	Do you have suggestions on how institutions could provide mental health support services for coping with stress related to this pandemic		Probes:<br>• How health system is prepared to address psychological needs of healthcare workers?<br>• How health system can ensure implementation of process and tolls to support mental health needs of healthcare workers?<br>• How health system will sustain the mental health support activities for its healthcare workers?
5.3	Do you have suggestions on how provincial/federal government could provide mental health support services for coping with stress related to this pandemic		

For peer review only - http://bmjopen.bmj.com/site/about/guidelines.xhtml

Table S1: Sample process of conventional content analysis

Text from transcript	Step 1: Readings transcripts and formulating open codes	Step 2: Developing Codebook	Step 3: Combined codes into categories	Step 4: Combine categories into themes
They (HCPs) were afraid for their families that our parents are old and if we carry coronavirus from here then we might not get them infected. Even if we don't show any symptoms, we would get them infected or we would get our children infected. So this fear was also prevailing	Infection to old parents<br><br>Infection to children	Fear of infection	Fear of of infection, isolation and stigma	Psychological impact of Covid-19 on health service providers
Immediately after that, there was fear of isolation, like if you put yourself into isolation who will look after your family and your kids …. Health workers were hospital-bound and were afraid of living alone and being cut off from their families. They also felt the stigma from the people around them	Afraid of living alone<br><br>No one to look after family<br><br>Stigma from the people	Fear of Isolation<br><br>Fear of Stigma		
Firstly, the PPEs we were using, we had to use them twice or thrice. this was our main issue. We had to wear the mask for two to three days even one week continuously<br><br>One can call it anxiety, you can see it, and they were under a depressive state. All healthcare workers were. From doctors to nursing staff, paramedics, our sweepers, all our workers were going through depression	Reuse PPEs<br><br>Workers going through depression	Lack of availability of PPEs<br><br>Depression due to COVID-19	Stress due to poor availability of personal protective equipment<br><br>Anxiety due to uncertainty of Covid-19	

For peer review only
